# *Pseudomonas virulence factor* controls expression of virulence genes in *Pseudomonas entomophila*

**DOI:** 10.1371/journal.pone.0284907

**Published:** 2023-05-18

**Authors:** Katie A. Acken, Bo Li

**Affiliations:** 1 Department of Chemistry, The University of North Carolina at Chapel Hill, Chapel Hill, North Carolina, United States of America; 2 Department of Microbiology and Immunology, The University of North Carolina at Chapel Hill, Chapel Hill, North Carolina, United States of America; Università degli Studi Roma Tre Dipartimento di Scienze: Universita degli Studi Roma Tre Dipartimento di Scienze, ITALY

## Abstract

Quorum sensing is a communication strategy that bacteria use to collectively alter gene expression in response to cell density. Pathogens use quorum sensing systems to control activities vital to infection, such as the production of virulence factors and biofilm formation. The *Pseudomonas virulence factor* (*pvf*) gene cluster encodes a signaling system (Pvf) that is present in over 500 strains of proteobacteria, including strains that infect a variety of plant and human hosts. We have shown that Pvf regulates the production of secreted proteins and small molecules in the insect pathogen *Pseudomonas entomophila* L48. Here, we identified genes that are likely regulated by Pvf using the model strain *P*. *entomophila* L48 which does not contain other known quorum sensing systems. Pvf regulated genes were identified through comparing the transcriptomes of wildtype *P*. *entomophila* and a *pvf* deletion mutant (Δ*pvfA-D*). We found that deletion of *pvfA-D* affected the expression of approximately 300 genes involved in virulence, the type VI secretion system, siderophore transport, and branched chain amino acid biosynthesis. Additionally, we identified seven putative biosynthetic gene clusters with reduced expression in Δ*pvfA-D*. Our results indicate that Pvf controls multiple virulence mechanisms in *P*. *entomophila* L48. Characterizing genes regulated by Pvf will aid understanding of host–pathogen interactions and development of anti-virulence strategies against *P*. *entomophila* and other *pvf*-containing strains.

## Introduction

Quorum sensing is a form of bacterial cell-to-cell communication that regulates collective behaviors in response to changes in cell density through the secretion and detection of signaling molecules called autoinducers [1, 2]. Quorum sensing influences biofilm formation [3, 4] as well as the production of small molecules [5] and virulence factors [6]. Some virulence factors are toxins, while others enable pathogens to evade host defenses [7], colonize and persist in the host, and outcompete other microbes [8]. Characterization of virulence factors and their regulation by quorum sensing is vital for understanding how bacteria cause infections and for developing anti-virulence strategies [9–12]. The pathogen *Pseudomonas aeruginosa* uses multiple quorum sensing systems to regulate gene expression and virulence [13], including those based on *N*-acyl homoserine lactones [14, 15] and 2-alkyl-4-quinolones [16–18]. However, many *Pseudomonas* spp., such as *Pseudomonas entomophila* L48, do not produce these quorum sensing molecules, limiting our understanding of signaling and virulence in these organisms [19].

*Pseudomonas entomophila* L48 kills *Drosophila melanogaster* larvae and adults after ingestion by persisting in the *Drosophila* gut and inflicting irreversible damage [20]. The oral infection route of *P*. *entomophila* provides a model for unraveling the mechanisms of *Drosophila* innate immunity, which can be extended to higher order eukaryotes [21]. *P*. *entomophila* uses a global regulatory system to control virulence—the GacS/GacA two-component signaling system (Gac) for which the signal remains unknown [22]. In 2010, a putative biosynthetic gene cluster (BGC), the *Pseudomonas virulence factor* (*pvf*), was found to be responsible for synthesizing unknown small molecules that are required for full virulence against *Drosophila* [23].

The *pvf* cluster encodes a nonribosomal peptide synthetase (PvfC), a non-heme diiron *N*-oxygenase (PvfB), and two proteins with unknown function (PvfA and PvfB) (Figure A in S1 Fig). We have shown in *P*. *entomophila* L48 that *pvf*-encoded enzymes synthesize extracellular small molecules that autoinduce *pvf* expression (PVF autoinducers). Expression of *pvf* in a reporter strain depends on cell density of the culture or the amount of cell-free supernatant added from cultures that express *pvfA*-*D*, which suggests quorum-sensing properties of the small molecules produced by *pvf*-encoded enzymes [24]. The genome of *P*. *entomophila* L48 does not harbor homologs of biosynthetic genes for autoinducers such as *N*-acyl homoserine lactones (*luxI*) or 2-alkyl-4-quinolones (*pqsABCDE*) [23, 25]. *P*. *entomophila* L48 is therefore a good model system for studying the Pvf signaling system (Pvf for short). Additionally, since the *pvf* cluster is conserved in more than 500 strains of proteobacteria, including the plant pathogen *Pseudomonas syringae* and the opportunistic human pathogen *Burkholderia cenocepacia* [26, 27], characterizing genes regulated by Pvf in *P*. *entomophila* has broad implications for understanding bacterial virulence and host–pathogen interactions in many different species.

The *pvf* homologs in *B*. *cenocepacia* H111 are part of the seven-gene *ham* cluster and encode enzymes HamA, HamC, HamD, and HamE, which exhibit 39–53% sequence identity to PvfA-D. The *ham* cluster (*hamA*-*G*) is responsible for synthesizing the diazeniumdiolate-containing antifungal compound fragin [28]; however, homologs of *hamB*, *hamF*, and *hamG* are not present in *P*. *entomophila* and the signaling molecules produced by *pvf*-encoded enzymes in *P*. *entomophila* are unknown.

Previously, we characterized the protein- and small molecule-secretome altered by Pvf in *P*. *entomophila* L48 using proteomic and metabolomic analyses [24]. Deletion of *pvfC* affected the abundance of many secreted proteins and secondary metabolites, and these effects were reversed by genetic complementation with the full *pvfA*-*D* cluster [24]. We found that Pvf regulates the production of nearly 200 secreted and membrane proteins including toxins and proteins involved in type VI secretion, motility, and siderophore transport, as well as the production of secondary metabolites such as the insecticidal labradorins, the antimicrobial pyreudiones, and many uncharacterized small molecules [24]. Pvf has also been linked to the expression of select genes in *P*. *entomophila* [23, 24, 29]. The variety of phenotypes [23, 24, 30] influenced by the Pvf signaling system warrants a genome-wide transcriptomic analysis.

Here we present a global analysis of genes regulated by Pvf in *P*. *entomophila* L48. We used RNA sequencing to quantify differences in gene expression between wildtype (WT) and a *pvf* deletion mutant (Δ*pvfA-D* or KO) (Figure B in S1 Fig) [24]. We found that Pvf upregulates the expression of more than 200 genes involved in a wide variety of pathways implicated in virulence, metabolism, and physiology. Furthermore, out of around 60 biosynthetic genes upregulated by Pvf, we identified seven putative BGCs that could produce novel secondary metabolites. Combined, these broad transcriptional changes propose a role for Pvf as a global virulence regulator in *P*. *entomophila* and highlight avenues for further study.

## Results

### Pvf regulates the expression of 301 genes

We sequenced RNA from wildtype and Δ*pvfA-D* strains of *P*. *entomophila* L48 and performed differential gene expression analysis. RNA samples were extracted from 24-hour stationary-phase cultures, when Pvf signaling is active [24]. The signaling activity of each culture was validated using a β-galactosidase reporter assay that quantifies the expression of monalysin, a pore-forming toxin and virulence factor regulated by Pvf [24, 29]. Expression of monalysin in the reporter strain was induced by the supernatant of wildtype culture but not by the supernatant of Δ*pvfA-D* culture (S2 Fig), which confirms the signaling activity of the wildtype culture and the lack of signaling activity of the Δ*pvfA-D* culture. The β-galactosidase reporter strains were not used for RNA-sequencing. Instead, RNA samples were extracted from wildtype and Δ*pvfA-D* cultures grown on two separate days (“batches”) to account for day-to-day variability [31], and 12 total RNA samples [32] were sequenced. High-quality sequencing reads and good coverage of protein-coding genes were obtained (Table 1, S1 Table, and S1 Dataset), and principal component analysis shows a difference between experimental groups and similarity amongst replicates (S3 Fig).

**Table 1:** Overview of RNA sequencing results for *P*. *entomophila* wildtype (WT) and Δ*pvfA-D* (KO) samples.

Genotype*	Avg. total reads	Avg. total mapped reads^a^	Avg. CDS mapped reads^b^	Avg. rRNA mapped reads^b^	Avg. tRNA & misc. RNA mapped reads^b^
WT [1]	27,879,677	27,001,303 (96.8%)	13,871,983 (51.4%)	117,503 (0.4%)	13,011,817 (48.2%)
WT [2]	30,194,213	29,176,713 (96.6%)	14,906,357 (51.1%)	98,817 (0.3%)	14,171,540 (48.6%)
Δ*pvfA-D* [1]	25,914,493	25,088,854 (96.8%)	12,142,087 (48.4%)	143,696 (0.6%)	12,803,071 (51.0%)
Δ*pvfA-D* [2]	30,398,273	29,311,627 (96.4%)	13,728,675 (46.8%)	123,190 (0.4%)	15,459,762 (52.7%)

CDS, DNA protein coding sequence.

* [1] and [2] indicate batch number.

^a^ Percentage shown for each sample group is average total mapped reads over average total reads.

^b^ Percentage shown for each sample group is average reads mapped to CDS, rRNA, or tRNA & miscellaneous RNA sequences over average total mapped reads.

**Fig 1:**
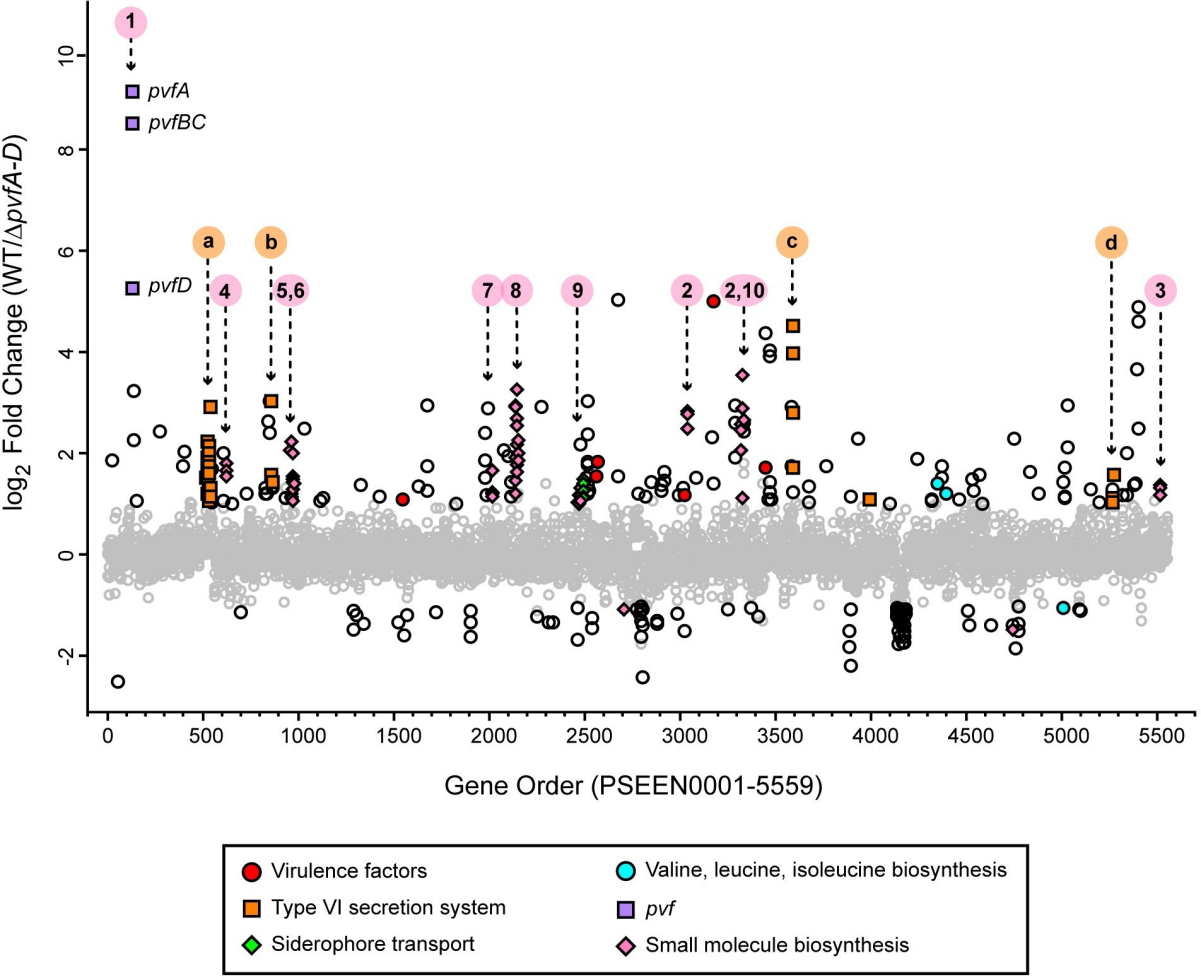
*entomophila* L48 wildtype (WT) cultures compared to Δ*pvfA-D*. Gene expression of *P*. Each point represents an annotated gene in the L48 genome with gene order (PSEEN0001-PSEEN5559) on the *x* axis and the log_2_ fold change of transcript abundance at 24 hrs in WT culture relative to Δ*pvfA-D* culture on the *y* axis. Differentially expressed genes are outlined in black (|log_2_ fold change| > 1 and *p* adjusted < 0.05). Point fill color and shape represents function: virulence factors (red circles), type VI secretion system (orange squares), siderophore transport (green diamonds), valine, leucine, and isoleucine biosynthesis (cyan circles), *pvf* (lilac squares), and small molecule biosynthesis (pink diamonds). Gene clusters with type VI secretion system-related functions further discussed in the text are annotated as (a) *P*. *entomophila* type VI secretion system locus (Pent-T6SS) and (b-d) group 1 orphan *vgrG*-containing clusters: PSEEN0861-0864 (b), PSEEN3592-3595 (c), and PSEEN5271, 5272, 5274–5276 (d). Putative small molecule biosynthetic gene clusters discussed in the text are labeled with the same cluster number as in Fig 5: cluster 1 (PSEEN0131-0134), cluster 2 (PSEEN3042-3045; PSEEN3332; PSEEN3335), cluster 3 (PSEEN5520-5522), cluster 4 (PSEEN0621-0624), cluster 5 (PSEEN0961-0972), cluster 6 (PSEEN0973-0987), cluster 7 (PSEEN2014-2021), cluster 8 (PSEEN2139-2144, 2146–2156), cluster 9 (PSEEN2466-2482), and cluster 10 (PSEEN3319-3331).

The genome of *P*. *entomophila* L48 contains 5,287 total annotated genes (5,134 protein coding genes, 78 tRNA genes, 22 rRNA genes, 10 miscellaneous RNA genes, 1 ncRNA gene, and 42 pseudogenes; S1 Dataset) of which 301 were differentially expressed between wildtype and Δ*pvfA-D* (*p* < 0.05 and fold change > 2; Fig 1). A total of 222 genes were upregulated and 79 were downregulated in wildtype compared with Δ*pvfA-D*, which demonstrates that Pvf affects about 6% of all genes. Most of these differentially expressed genes are protein-coding genes, although 4 pseudogenes, 1 rRNA-encoding gene, and the regulatory RNA PrrF1 were also differentially expressed. Gene functions were annotated based on information from NCBI RefSeq [33, 34], UniProt [35], the Pseudomonas Genome Database [36], and literature sources. The differentially expressed genes are likely regulated by Pvf and play a role in various processes, including branched chain amino acid biosynthesis, the type VI secretion system, siderophore transport, virulence, and biosynthesis of secondary metabolites (Fig 1).

### Pvf differentially regulates branched chain amino acid biosynthetic genes

**Fig 2:**
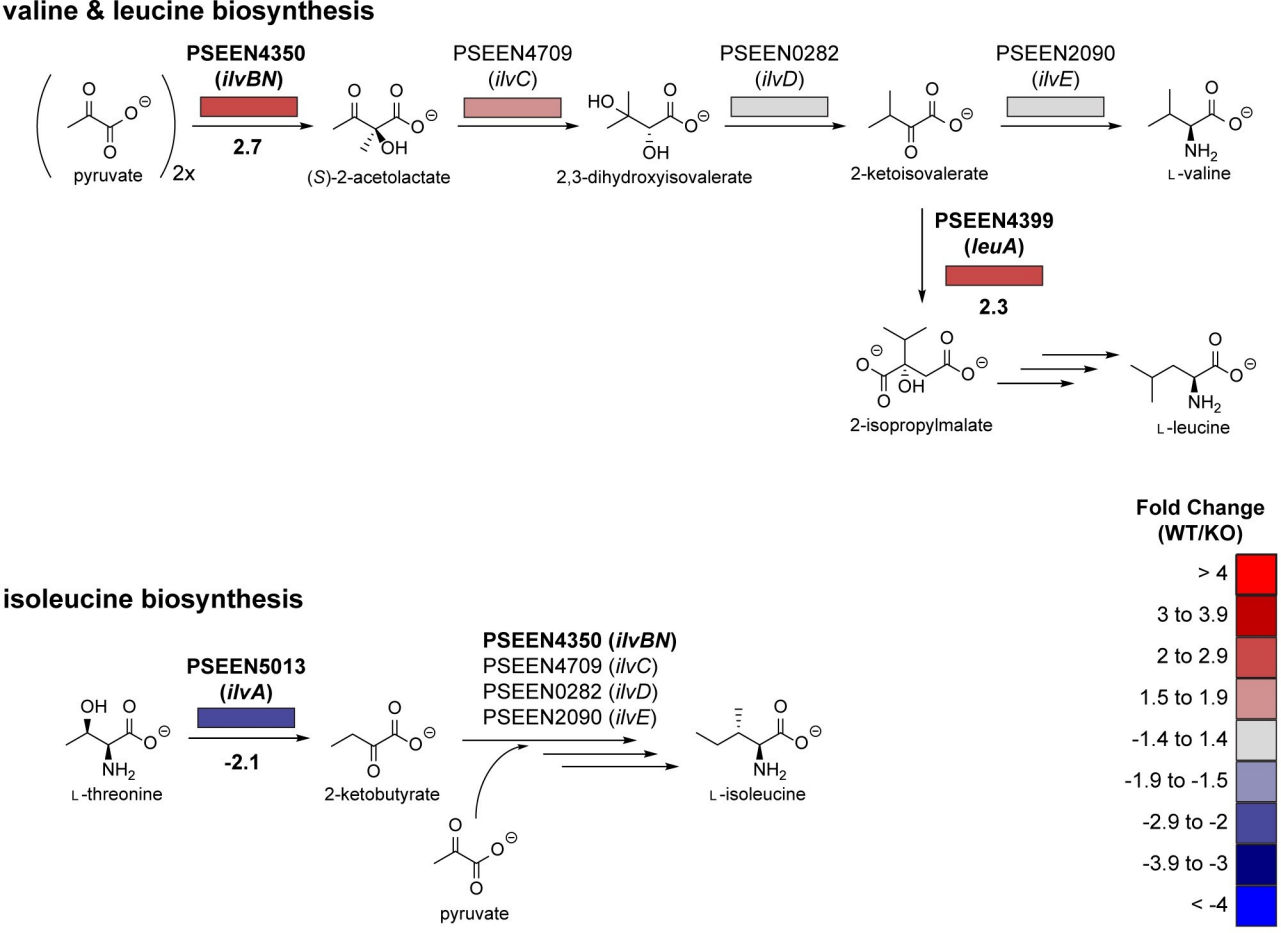
Genes in branched chain amino acid biosynthesis are differentially expressed between *P*. *entomophila* WT and Δ*pvfA-D* (KO). The general biosynthetic pathway for l-valine, l-leucine, and l-isoleucine in bacteria is shown with *Escherichia coli* genes (in parentheses) and their corresponding *P*. *entomophila* homologs. Boxes below genes represent fold change (FC) of transcript levels in WT relative to Δ*pvfA-D*, and FC values > 2 are shown. Transcripts expressed at higher levels in WT are depicted with increasingly dark shades of red, while transcripts expressed at higher levels in KO are depicted with increasingly dark shades of blue (bottom right). Negative fold change indicates transcripts expressed at higher levels in KO than WT and is calculated as fold change of KO relative to WT with a negative sign added. The same color scheme for fold change of transcript levels is used for all figures.

We observed different expression levels of genes required for the biosynthesis of the branched chain amino acids, l-valine, l-leucine, and l-isoleucine, between wildtype and Δ*pvfA-D* (Fig 2). The biosynthetic pathways of these amino acids are intertwined in bacteria and share four core enzymes—the acetolactate synthase IlvB/N, the keto acid reductoisomerase IlvC, the dihydroxyacid dehydratase IlvD, and the aminotransferase IlvE—which are sufficient to produce l-valine from two molecules of pyruvate [37]. The biosynthesis of l-isoleucine begins with an additional enzyme, the l-threonine dehydratase/deaminase IlvA, which converts l-threonine to 2-ketobutyrate [37]. A molecule of 2-ketobutyrate and a molecule of pyruvate are then processed by IlvB/N, IlvC, IlvD, and IlvE to generate l-isoleucine [37]. The biosynthesis of l-leucine parallels l-valine biosynthesis, then branches off by the action of the isopropylmalate synthase LeuA [37]. In wildtype compared with Δ*pvfA-D*, we observed higher expression (2.7-fold) of a gene shared in all three pathways–*ilvB* (PSEEN4350; Fig 2). Expression of *leuA* (PSEEN4399), only involved in l-leucine biosynthesis, was also higher (2.3-fold) in the wildtype (Fig 2). In contrast, expression of *ilvA* (PSEEN5013), the first gene in l-isoleucine biosynthesis from l-threonine, was lower (2.1-fold) in the wildtype than the mutant (Fig 2). These results suggest that Pvf upregulates l-valine and l-leucine biosynthesis but downregulates l-isoleucine biosynthesis.

### Pvf highly upregulates genes in the type VI secretion system

**Fig 3:**
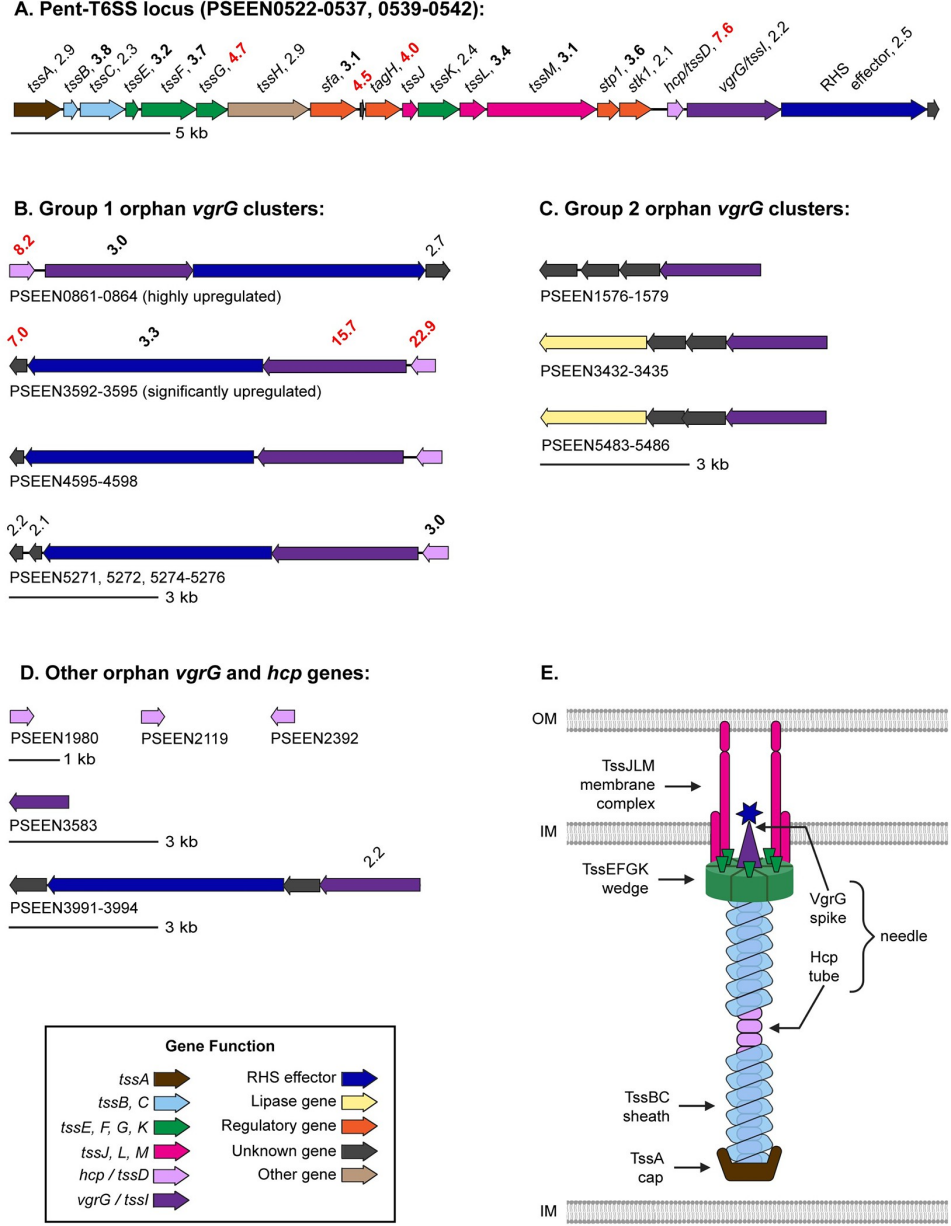
Genes in the type VI secretion system are differentially expressed between *P*. *entomophila* WT and Δ*pvfA-D* (KO). Arrows represent gene length and direction of transcription and are color-coded based on function: *tssA* (brown), *tssBC* (light blue), *tssEFGK* (green), *tssJLM* (pink), *hcp*/*tssD* (light purple), *vgrG/tssI* (dark purple), RHS effector (dark blue), lipase gene (yellow), regulation (orange), unknown function (grey), and other function (tan). Fold changes > 2 (with *p* < 0.05) of transcript levels in WT relative to Δ*pvfA-D* are displayed above the corresponding gene. Fold change values ≥ 3 are bolded and values ≥ 4 are red. Fold changes and *p* values for all genes shown in the figure can be found in Tab 4 of S1 Dataset. (A) The only T6SS locus in *P*. *entomophila*, Pent-T6SS, which encodes essential structural proteins. (B-D) Orphan *vgrG* and *hcp* genes are grouped based on phylogeny (S4 Fig) as follows: (B) group 1, (C) group 2, and (D) other. (E) Model of T6SS adapted from Cherrak *et al*. [38]. Proteins are colored to match their corresponding genes (bottom left).

Type VI secretion system (T6SS) genes constitute the largest category of genes upregulated by Pvf (Fig 1). The T6SS is a nano syringe in Gram-negative bacteria that injects toxic effector proteins into target cells [38] (Fig 3E). A large variety of effectors can be delivered by the T6SS [39, 40], including antibacterial proteins for interbacterial competition [8, 41] and eukaryotic-targeting virulence factors [42]. Pathogens typically encode multiple T6SS loci, which contain all 13 core genes (*tssA−M*) [43]. However, *P*. *entomophila* contains only one locus (PSEEN0522−0537, 0539−0542; Pent-T6SS; Fig 3A), which was highly expressed in the wildtype compared to Δ*pvfA-D* (Fig 3A). For example, transcripts of *tssM* (PSEEN0535) and *tssL* (PSEEN0534) that encode membrane complex proteins were more abundant in the wildtype (3.1- and 3.4-fold, respectively), and transcripts of genes that encode wedge proteins (*tssEFGK*; PSEEN0525−0527 and PSEEN0533) were between 2.4 and 4.7-fold higher in the wildtype (Fig 3A). The membrane complex and wedge together orient the needle, which consists of a spike complex formed by the trimeric valine-glycine repeat protein (VgrG or TssI) and a hollow tube formed by hexameric rings of haemolysin co-regulated protein (Hcp or TssD). We observed 2.2-fold higher gene expression of the T6SS locus *vgrG* (PSEEN0540) and 7.6-fold higher expression of the T6SS locus *hcp* gene (PSEEN0539) in the wildtype (Fig 3A). A sheath composed of proteins encoded by *tssB* (PSEEN0523) and *tssC* (PSEEN0524) wraps the shaft and upon contraction launches the needle into the host cell. Transcript levels of *tssB* and *tssC* were 3.8-fold and 2.3-fold higher in the wildtype, respectively (Fig 3A). The wildtype also exhibited 2.9-fold higher expression of *tssA* (PSEEN0522), which encodes a cap protein that mediates polymerization of the Hcp tube and sheath, and 2.9-fold higher expression of *tssH* (PSEEN0528), which encodes an ATPase involved in recycling the contracted sheath (Fig 3A). Additionally, transcripts of three type VI secretion system regulatory genes were more abundant in the wildtype—PSEEN0529 (sigma factor activator, *sfa*, 3.1-fold higher), PSEEN0536 (phosphoprotein phosphatase *stp1*, 3.6-fold higher), and PSEEN0537 (serine-threonine protein kinase *stk1*, 2.1-fold higher; Fig 3A). Overall, higher transcript levels of all T6SS core components in the wildtype suggest significant upregulation of the Pent-T6SS by Pvf.

In addition to the *vgrG* and *hcp* genes in the T6SS locus, multiple additional “orphan” *vgrG* and *hcp* genes outside of the main T6SS locus (Fig 3B–3D) were expressed at a higher level in the wildtype than Δ*pvfA-D*. These orphan *vgrG* genes can be separated into 2 distinct groups based on sequence identity and neighboring genes [44, 45]. Group 1 *vgrG* genes share greater than 81% pairwise amino acid sequence identity with each other and with the PSEEN0540 *vgrG* in the main T6SS locus (S4 Fig). The high sequence similarity suggests that group 1 *vgrG*s are inparalogs [44], or copies, of the PSEEN0540 *vgrG* and might perform similar functions. Group 1 *vgrG* genes have an adjacent *hcp* gene and an adjacent RHS-repeat containing effector gene (Fig 3B). Furthermore, the C-terminus of all group 1 *vgrG* genes includes DUF2345, which has been linked to recruitment of effectors in *E*. *coli* [46] as well as antibacterial and antieukaryotic activity in *Klebsiella pneumoniae* [47]. Among the group 1 *vgrG* genes, we observed higher expression of PSEEN0862 and PSEEN3594 (3.0- and 15.7-fold, respectively) in the wildtype (Fig 3B). Transcripts of multiple genes that are adjacent to group 1 *vgrG* genes were also more abundant in the wildtype (Fig 3B), including the *hcp* genes PSEEN0861, PSEEN3595, and PSEEN5276 (8.2-, 22.9-, and 3.0-fold higher, respectively) and the RHS effector gene PSEEN3593 (3.3-fold higher). Four other highly expressed genes adjacent to group 1 *vgrG* genes (PSEEN0864, PSEEN3592, PSEEN5271, and PSEEN5272; 2.7-, 7.0-, 2.2-, and 2.1-fold higher in WT than Δ*pvfA-D*, respectively) are not annotated and may encode T6SS effectors or immunity proteins. Therefore, Pvf upregulates group 1 *vgrG*s and their associated genes.

The group 2 *vgrG* genes share greater than 62% pairwise amino acid sequence identity amongst themselves (S4 Fig). Instead of being adjacent to *hcp* and RHS effector genes, group 2 *vgrG*s are nearby genes that encode lipase effectors and uncharacterized proteins (Fig 3C). None of the group 2 *vgrG* genes or neighboring genes were differentially expressed between wildtype and Δ*pvfA-D* in our dataset (Fig 3C and S1 Dataset). Outside of group 1 and 2 *vgrG*s, the genome of *P*. *entomophila* contains two additional orphan *vgrG* genes, which exhibit relatively low sequence identity with each other and with the other *vgrG* genes (Fig 3D and S6 Fig). One of these *vgrG* genes, PSEEN3994, was expressed at a 2.2-fold higher level in the wildtype (Fig 3D). Therefore, Pvf differentially upregulates the expression of this orphan *vgrG* gene.

### Pvf upregulates genes involved in siderophore transport

**Fig 4:**
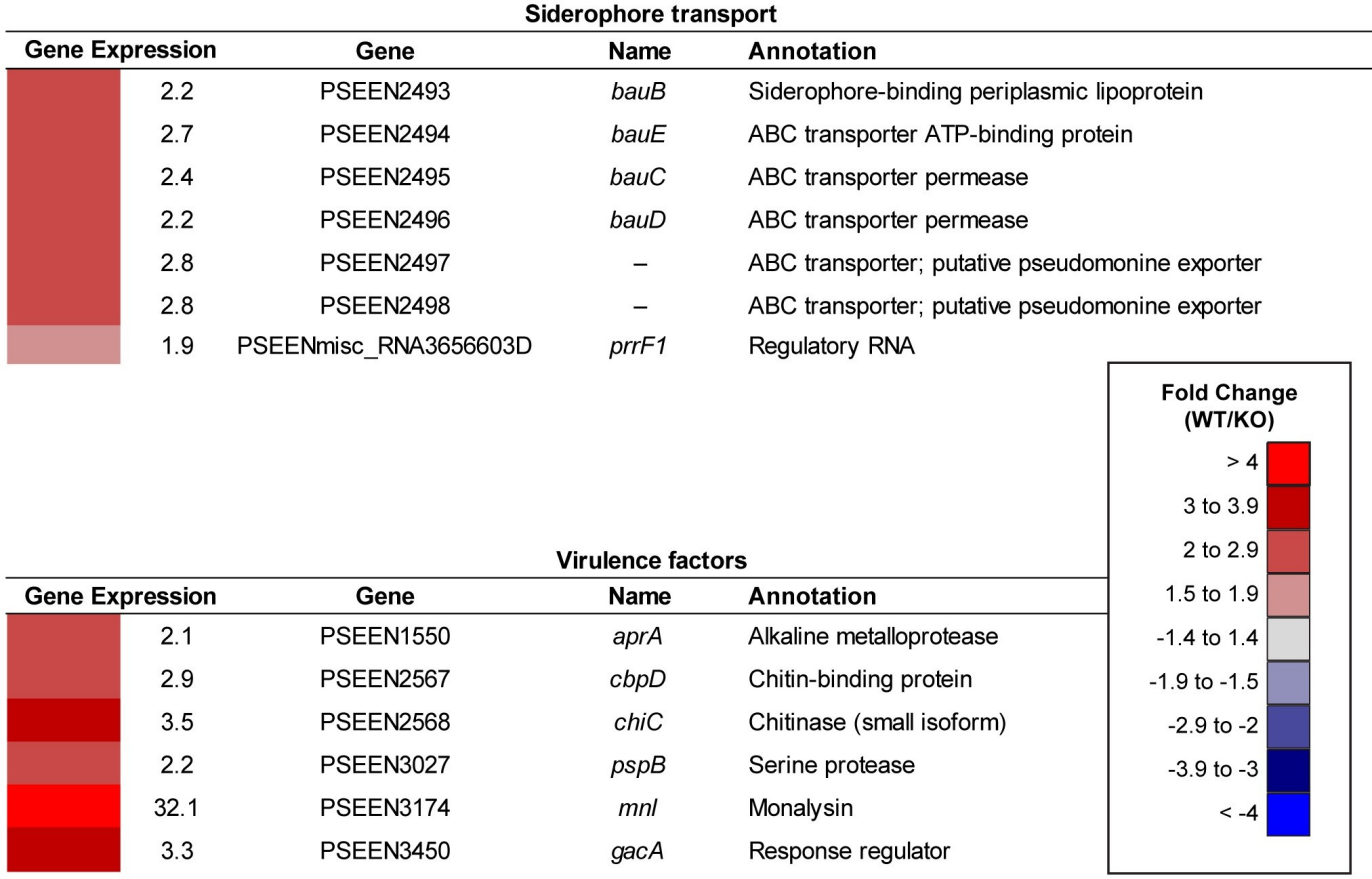
Additional genes differentially expressed between *P*. *entomophila* WT and Δ*pvfA-D* (KO). Numerical values represent fold change of transcript levels in WT relative to Δ*pvfA-D* (KO). Only genes with *p* value < 0.05 are shown. Annotations adapted from NCBI and the Pseudomonas Genome Database.

Several genes involved in siderophore transport were expressed at significantly different levels between wildtype and Δ*pvfA-D* (Fig 4), although no significant changes were observed in expression of siderophore biosynthetic genes (S1 Dataset). *P*. *entomophila* produces at least two siderophores, a pyoverdine and pseudomonine [48, 49]. Pseudomonine is structurally similar to acinetobactin from *Acinetobacter baumannii* [50], and likely uses a TonB-dependent import system that is homologous to *bauABDCE* in acinetobactin import [49, 51]. Wildtype exhibited higher gene expression levels than Δ*pvfA-D* of the *bauB* (PSEEN2493, 2.2-fold), *bauC* (PSEEN2495, 2.4-fold), *bauD* (PSEEN2496, 2.2-fold), and *bauE* (PSEEN2494, 2.7-fold) homologs, suggesting that Pvf upregulates ferric pseudomonine uptake (Fig 4). Furthermore, transcript levels of two ABC transporter-encoding genes (PSEEN2497 and PSEEN2498) that may be involved in pseudomonine export were 2.8-fold higher in the wildtype compared to Δ*pvfA-D* (Fig 4). We also observed 1.9-fold higher levels of a homolog of *prrF1* in the wildtype (Fig 4), which encodes a small regulatory RNA required for iron homeostasis and virulence in *P*. *aeruginosa* [52, 53].

### Pvf upregulates additional virulence factor genes

Many genes that encode virulence factors such as proteases, lipases, chitinases, and toxins are differentially expressed between the wildtype and Δ*pvfA-D* (Fig 4). These virulence factors are used by bacteria to overcome insect defenses [54]. Expression of the gene encoding the virulence factor monalysin (*mnl*, PSEEN3174), which causes irreversible damage to the *D*. *melanogaster* gut epithelium [29], is significantly higher (32.1-fold; Fig 4) in the wildtype than Δ*pvfA-D*, making it the 6^th^ most Pvf-upregulated gene overall (Fig 1 and S1 Dataset). Additionally, transcripts of protease genes *aprA* (PSEEN1550) and *pspB* (PSEEN3027) were more abundant in the wildtype than Δ*pvfA-D* (2.1- and 2.3-fold, respectively; Fig 4). The *aprA* gene encodes a metalloprotease responsible for activating monalysin by cleaving the N-terminus of the inactive pre-monalysin [29], and further contributes to pathogenicity and persistence by degrading antimicrobial peptides secreted by the *D*. *melanogaster* gut epithelium [21]. Transcript abundance of the chitinase-encoding gene *chiC* (PSEEN2568) was significantly higher (3.5-fold) in the wildtype than Δ*pvfA-D* (Fig 4). Chitinase activity of *chiC*, a gene shared amongst highly insecticidal members of the *Pseudomonas fluorescens* group, has been linked to the oral insecticidal activity of *Pseudomonas protegens* CHA0 [55]. We also observed higher expression (2.9-fold) in the wildtype of a homolog of *cbpD* (chitin-binding protein D, PSEEN2567; Fig 4), which encodes a lytic polysaccharide monooxygenase. CbpD oxidizes chitin [56] and promotes survival of *P*. *aeruginosa* PA7 during systemic infection in humans [57]. A protective layer of chitin and glycoproteins coats the insect intestinal lumen [58], and the proteins encoded by *chiC* and *cbpD* could work together to break down this barrier. Finally, *gacA* (PSEEN3450), the response regulator in the GacS/A two-component system, was more highly expressed in wildtype than Δ*pvfA-D* (3.3-fold; Fig 4).

### Pvf upregulates the expression of ten biosynthetic gene clusters

**Fig 5:**
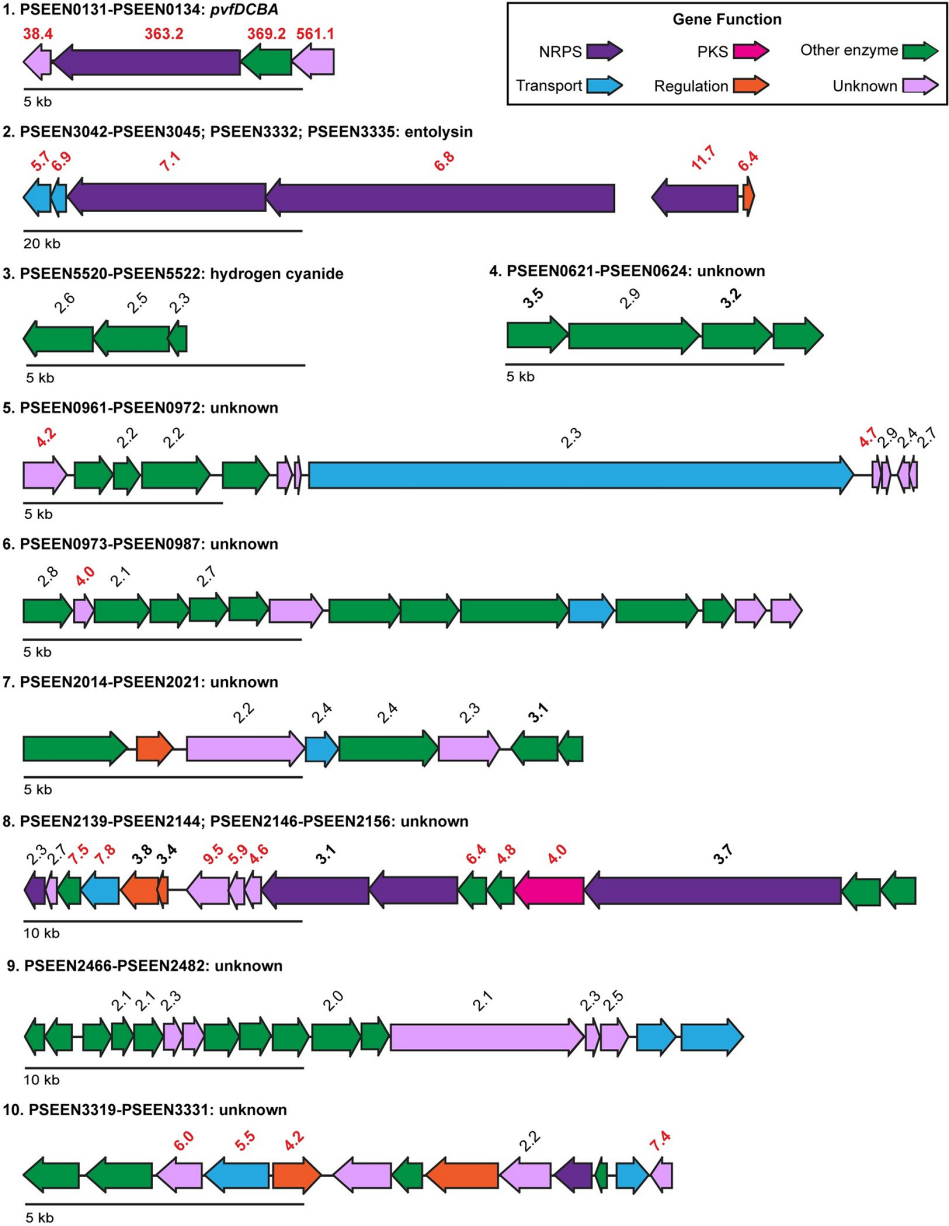
Biosynthetic gene clusters differentially expressed between *P*. *entomophila* WT and Δ*pvfA-D*. Arrows represent gene length and direction of transcription and are color-coded based on function: NRPS (dark purple), PKS (pink), other enzyme (green), transport (aqua), regulation (orange), and unknown (light purple). Fold changes > 2 (with *p* < 0.05) of transcript levels in WT relative to Δ*pvfA-D* are displayed above the corresponding gene. Fold change values ≥ 3 are bolded and values ≥ 4 are red. Fold changes, *p* values, and putative enzyme functions for all genes shown in the figure can be found in Tab 7 of S1 Dataset.

Transcripts for many secondary metabolite biosynthetic genes were more abundant in the wildtype than Δ*pvfA-D*, which suggests that Pvf upregulates their expression (Fig 5). As expected, expression of the *pvf* biosynthetic gene cluster (Fig 5, cluster 1) is diminished in the Δ*pvfA-D* strain. Two additional gene clusters that produce known secondary metabolites were also expressed at an elevated level in the wildtype compared to Δ*pvfA-D*. For example, all genes involved in entolysin biosynthesis, regulation, and transport (Fig 5, cluster 2) were expressed at significantly higher levels in the wildtype (> 4-fold), which indicates that Pvf controls entolysin production at the transcriptional level. Additionally, the genes responsible for hydrogen cyanide biosynthesis (*hcnCBA*; Fig 5, cluster 3) were expressed at levels 2.3 to 2.6-fold higher in the wildtype, which suggests that Pvf upregulates the production of hydrogen cyanide, which is also a virulence factor.

We also identified seven putative biosynthetic gene clusters (BGCs) based on increased expression of constituent genes in the wildtype compared to Δ*pvfA-D* (Fig 5). For example, three of four genes in cluster 4 (PSEEN0621-0624) were expressed 2.9- to 3.5-fold higher in the wildtype than in Δ*pvfA-D* (Fig 5; S1 Dataset). A search of cluster 4 against the GenBank database using MultiGeneBlast [59] revealed homologous clusters in *Yersinia* and *Klebsiella* species, but not in other pseudomonads (S5 Fig). Genes in another putative BGC, cluster 5 (Fig 5; PSEEN0961−PSEEN0972), exhibited 2.2- to 4.7-fold higher expression in the wildtype (S1 Dataset). While other strains of *Pseudomonas* contain individual genes homologous to those in cluster 5, this cluster as a whole is not well conserved in pseudomonads (S6 Fig). Genes PSEEN0973−PSEEN0987 form another potential BGC (Fig 5, cluster 6) and these genes were expressed between 2.1- and 4-fold higher in the wildtype (S1 Dataset). Similar clusters to cluster 6 exist in the nematode symbiont and insect pathogen *Xenorhabdus nematophila* (S7 Fig). Cluster 7 (Fig 5; PSEEN2014−PSEEN2021) includes five genes with 2.2- to 3.1-fold higher expression in the wildtype (S1 Dataset). The closest relatives to cluster 7 exist in *A*. *baumannii*, and no homologous clusters were found in pseudomonads (S8 Fig).

Transcripts of most genes from cluster 8 (Fig 5; PSEEN2139−2144, 2146−2156) were at least 3-fold more abundant (and many > 4-fold) in the wildtype (S1 Dataset), including genes that encode three nonribosomal peptide synthetases (NRPSs; PSEEN2139, PSEEN2149, PSEEN2154) and one polyketide synthase (PKS; PSEEN2153) (2.3-, 3.1-, 3.7-, and 4.0-fold higher, respectively). NRPSs and PKSs are major enzyme families that synthesize secondary metabolites; NRPS/PKS hybrid clusters can synthesize peptide and polyketide hybrid products, such as lipopeptides [60]. Additional enzyme-encoding genes in the NRPS/PKS hybrid cluster 8 include a putative *O*-methyltransferase gene (PSEEN2141; 7.5-fold more highly expressed in the wildtype), the uncharacterized gene (PSEEN2146; 9.5-fold higher), and a putative ornithine cyclodeaminase gene (PSEEN2151; 6.4-fold higher; Fig 5 and S1 Dataset). Expression of an efflux pump gene (PSEEN2142), potentially involved in secondary metabolite export, was 7.8-fold higher in the wildtype than Δ*pvfA-D* (Fig 5 and S1 Dataset). These data suggest that Pvf upregulates the secondary metabolites produced by cluster 8-encoded enzymes. A search of cluster 8 against the GenBank database conducted with MultiGeneBlast [59] revealed only one homologous cluster in the phytopathogen *Burkholderia gladioli* BSR3 (S9 Fig).

Potential exopolysaccharide cluster 9 (Fig 5; PSEEN2466−PSEEN2482) contains several uncharacterized genes with higher transcript levels in wildtype (2.0- to 2.5-fold; S1 Dataset) and appears unique to *P*. *entomophila* (S10 Fig). Finally, we observed significantly higher expression in the wildtype (> 4-fold; S1 Dataset) of four genes in cluster 10 (Fig 5; PSEEN3319−PSEEN3331). A few strains of *Pseudomonas* contain sets of genes homologous to those in cluster 10, otherwise this cluster as a whole is not well conserved in pseudomonads (S11 Fig). Activities of the enzymes encoded in clusters 4 through 10 and structures of the secondary metabolites produced by these enzymes warrant further investigation. Pvf control of these seven proposed BGCs indicate the untapped biosynthetic potential of *P*. *entomophila* that may be explored using Pvf as a guide.

## Discussion

Here we characterized the transcriptome of the Pvf signaling system in *P*. *entomophila* L48 by comparing gene expression between wildtype and a *pvfA-D* mutant. We found that deletion of *pvfA-D* controls the expression of genes in numerous pathways including branched chain amino acid biosynthesis, the type VI secretion system, siderophore transport, virulence, and biosynthesis of secondary metabolites. We did not further analyze genes that exhibited less than 2-fold difference in expression between wildtype and the *pvfA-D* mutant in our dataset, but expression of some of these genes could also be affected by Pvf.

Our transcriptomics suggest that Pvf upregulates l-valine (*ilvBN*) and l-leucine (*ilvBN* and *leuA*) biosynthesis and downregulates l-isoleucine biosynthesis (*ilvA*) in *P*. *entomophila* L48. Similarly, the *ham* cluster in *B*. *cenocepacia* H111 that contains *pvf* homologs upregulates an *ilvD* homologue and two *leuA* homologs, which also suggests upregulation of l-valine and l-leucine biosynthesis [28]. l-Valine and l-leucine could then serve as starting materials for *pvf or ham*-encoded enzymes. These amino acids are precursors to several small molecules including the dihydropyrazine *N*-oxides [27, 61] and leucinazole [62], which are synthesized by *pvf*-encoded enzymes in *P*. *entomophila and P*. *fluorescens* Pf0-1. l-Valine is the precursor to diazeniumdiolate-containing compounds valdiazen and fragin, which are produced by *ham*-encoded enzymes in *Burkholderia cenocepacia* [63].

The T6SS may play an important role in *P*. *entomophila* virulence, since unlike many other Gram-negative pathogens it does not encode a type III secretion system (T3SS). T6SS loci in *Pseudomonas* spp. cluster into six distinct phylogenetic groups that correlate with function [45]. The *P*. *entomophila* T6SS (Pent-T6SS) belongs to the Hcp secretion island two (HSI-II) cluster, which has been linked to virulence in *Burkholderia thaliandensis* [64] and plant and animal virulence in *P*. *aeruginosa* [65]. As the sole T6SS locus, the Pent-T6SS is a good model to investigate the role of the HSI-II type T6SS in infection and virulence. Co-regulation of *vgrG* genes with Pent-T6SS components suggests that the Pent-T6SS may be responsible for secreting the group 1 VgrG proteins and putative effector proteins encoded nearby. In contrast, group 2 *vgrG*s do not appear to be co-regulated with the Pent-T6SS, which suggests that group 2 *vgrG*s might perform a T6SS-independent function. Compared to other bacterial secretion systems, only a limited number of T6SS effectors and immunity proteins have been identified to date [66]. Here, we identified a number of putative T6SS effectors or immunity proteins highly upregulated by Pvf, many of which contain uncharacterized domains, like DUF2345. Our work paves the way for further studying the functions of these proteins.

Results from transcriptional analysis mostly agree with previous analysis of the secreted proteome and yielded new insights on Pvf regulation. For example, Pvf upregulates secretion of the virulence factors AprA and monalysin as well as VgrG spike proteins and RHS effector proteins encoded by the Pent-T6SS locus and group 1 *vgrG* clusters [24]. The corresponding genes also showed higher expression levels in the WT than the *pvfA-D* mutant. However, Pvf was linked to reduced swarming motility and decreased secretion of flagellar proteins by proteomic and phenotypic analysis [24], but differential expression of the corresponding genes was not captured in the transcriptomic analysis. This is not surprising because transcriptomic and proteomic datasets often only exhibit modest correlation due to differences in methodology and regulatory mechanisms (i.e. changes in protein abundance may result from post-transcriptional or -translational regulation or degradation) [67, 68]. New insights on Pvf regulation from transcription analysis include upregulation of branched chain amino acid metabolism and a putative hybrid sensory box/response regulator, the latter of which has a close ortholog in *Pseudomonas putida* (83% amino acid sequence identity) that has been linked to increased c-di-GMP levels, decreased motility, and increased biofilm formation [69, 70]. How Pvf regulation is linked to c-di-GMP signaling in *P*. *entomophila* will require further investigation. Altogether, transcriptomics and proteomics offered complementary insights on Pvf regulation.

Deletion of *pvfA-D* reduced the expression of a number of genes involved in pseudomonine transport, which could suggest a preference by Pvf for upregulating pseudomonine uptake over other iron-import systems. Interestingly, expression of genes involved in pseudomonine and pyoverdine biosynthesis do not appear significantly altered by Pvf. Perhaps, to conserve metabolic resources during infection, Pvf increases pseudomonine transport rather than production. Furthermore, *P*. *entomophila* has been shown to effectively import exogenous pyoverdine siderophores [49], so the pseudomonine uptake pathway may allow for import of structurally similar siderophores.

*P*. *entomophila* produces many different secondary metabolites, including hydrogen cyanide, the pyreudiones, and the labradorins [24]. Our data indicate that Pvf upregulates hydrogen cyanide on the transcriptional level. *P*. *entomophila* is one of a few known hydrogen cyanide producers, but Pvf had not been linked to regulation of this activity [71]. Hydrogen cyanide is known to be a virulence factor in *Pseudomonas*, including *P*. *aeruginosa*, where it is transcriptionally regulated by the *las* and *rhl* quorum sensing systems [72]. We did not observe statistically significant trends in transcript levels of pyreudione and labradorin biosynthetic genes in wildtype or Δ*pvfA-D*, although metabolomics results indicate that Pvf upregulates production of these metabolites [24]. It is possible that regulation of these metabolites occurs post-transcriptionally or that Pvf-induced transcriptional changes were not captured in this dataset. Furthermore, our previous metabolomics analysis revealed that the presence of Pvf increases the production of many uncharacterized metabolites [24]. Here, we identified seven putative BGCs that are transcriptionally upregulated by Pvf, which might correspond to increased production of these uncharacterized metabolites. In addition, many of these putative BGCs are unique to *P*. *entomophila* and encode uncharacterized proteins. Therefore, we anticipate that Pvf can be used to elicit production of novel secondary metabolites for structural elucidation and enable characterization of new enzymes involved in their biosynthesis.

Genes affected by Pvf exhibit some overlap with those affected by the GacS/A system, a two-component signal transduction system that is conserved in Gram-negative bacteria. In response to an unknown signal at high cell densities, Gac regulates numerous genes involved in virulence, quorum sensing, motility, stress tolerance, and biofilm formation [73, 74]. In pseudomonads, Gac regulates genes involved in virulence factor production, type II/III/VI secretion systems, siderophore biosynthesis and export, and small molecule biosynthesis [75, 76]. In *P*. *entomophila*, Gac has been found to upregulate the production of monalysin, the protease AprA, hydrogen cyanide, entolysin, and some T6SS components [22, 29]. Despite considerable overlap in target genes, Pvf and Gac regulatory mechanisms are proposed to operate independently [23]. However, our finding that Pvf upregulates gene expression of *gacA* raises the possibility of cross talk between the two regulatory systems.

In conclusion, the RNA-sequencing data indicate that Pvf controls the transcription of more than 300 genes. These findings demonstrate the broad regulatory effects of the Pvf signaling system on the physiology and virulence of *P*. *entomophila* L48. We identify many putative virulence factors under Pvf control whose functions are unknown and potentially novel. Further characterization of these virulence factors will contribute to understanding the mechanisms of virulence and host–pathogen interactions in *P*. *entomophila* and other pseudomonads. Furthermore, the large number of biosynthetic gene clusters upregulated by Pvf suggests that Pvf regulation may be used to identify novel small molecules produced by this bacterium. The receptor and signal transduction pathway of Pvf will be a focus of future studies.

## Materials and methods

### Source of chemicals and biological reagents

Chemical reagents were purchased from Sigma Aldrich or ThermoFisher Scientific unless otherwise stated.

### Bacterial strains and culture conditions

All *P*. *entomophila* L48 strains were cultured in Lennox Luria Broth (low salt LB or LSLB) at 30°C with 225 rpm shaking, unless otherwise stated. The markerless *pvf* deletion mutant in *P*. *entomophila*, Δ*pvfA-D*, was constructed previously [24] and only includes the final 213 bp of *pvfD* (less than half of the original 468 bp). The previously constructed β-galactosidase assay reporter strains *P*. *entomophila* Δ*pvfC*::attTn7-P*mnl*-*lacZ* and WT::attTn7-P*mnl*-*lacZ* [26] contain a kanamycin resistance marker, and kanamycin was added to cultures of these strains to a final concentration of 50 μg mL^-1^.

### Cell culture and RNA extraction for RNA sequencing

Flasks of 25 mL cultures of *P*. *entomophila* L48 wildtype and Δ*pvfA-D* were inoculated with 50 μL of overnight culture and grown for 24 hr. Three biological replicates each of wildtype and Δ*pvfA-D* were grown. After 24 hr, 1 mL of each culture was removed for RNA extraction and the remaining 24 mL of culture was saved and tested for PVF autoinducer signaling activity (see below). For RNA extraction, cells were harvested by centrifugation for 30 min at 4°C, 3500 x g. RNA was extracted with TRIzol (ThermoFisher Scientific), as per the manufacturer’s protocol. DNase treatment was immediately performed with TURBO DNase (Invitrogen) as follows: 42 μL of nuclease-free water (NFW), 5 μL of TURBO DNase buffer, and 2 μL of TURBO DNase were added to each sample. Samples were incubated at 37°C for 30 min, then 1 μL of TURBO DNase was added, and samples were incubated for an additional 30 min at 37°C. RNA was purified with Mag-Bind TotalPure NGS beads (Omega Bio-tek) as per the manufacturer’s protocol. In brief: 90 μL of beads were added to each 50 μL DNase reaction (1.8X ratio). Samples were incubated for 5 min then transferred to a magnetic rack and incubated an additional 5–8 min. Samples were washed three times with 200 μL of 70% ethanol, then air dried for 5–8 min. RNA was eluted into 50 μL of nuclease-free water and stored at −80°C. A second batch of cultures was grown at a later time and RNA extraction repeated, resulting in a second batch of RNA samples. RNA quality was assessed using the Agilent BioAnalyzer2100, and all samples had a RIN > 8 and a 23S/16S ratio ≥ 1.2.

### RNA sequencing and data analysis

A total of 12 RNA samples were submitted to BGI Genomics for sequencing (6 wildtype and 6 Δ*pvfA-D*). Vazyme Ribo-off rRNA removal kit was used for rRNA depletion. The BGI DNBSEQ-G400 platform was used for library construction and sequencing. Libraries were non-stranded and 100 bp paired-end reads were obtained. BGI removed adapters, contamination, and poor-quality reads; read statistics (S1 Table) represent read quality after the filtering by BGI. Reads were aligned to the *Pseudomonas entomophila* L48 genome using bbmap [77]. The reference genome was obtained from the NCBI RefSeq Database (accession number: GCF_000026105.1) [33] and the GTF annotation file was obtained from the Pseudomonas Genome Database [36]. The program featureCounts from the subread package was used to generate the count table [78]. Differential expression and statistical analysis were performed with DESeq2 [79]. The log_2_ fold changes (WT/KO) were mapped onto biological pathways from the Kyoto Encyclopedia of Genes and Genomes (KEGG) Database [80] using the KEGGParser [81] application in Cytoscape [82]. Multiple sequence alignments were performed with Clustal Omega [83]. Gene cluster homology searches were conducted with MultiGeneBlast [59] against the 12/2015 version of the GenBank genome database.

### PVF autoinducer signaling activity assay

The remaining 24 mL of each WT and Δ*pvfA-D* culture (after aliquots were removed for RNA extraction) was centrifuged for 40 min at 4°C, 4500 x g, then the supernatant transferred into a fresh tube and stored at 4°C. Next, the PVF autoinducer signaling activity of the WT and Δ*pvfA-D* culture supernatants was tested in a β-galactosidase reporter assay (β-gal assay) with the Δ*pvfC*::attTn7-P*mnl*-*lacZ* and WT::attTn7-P*mnl*-*lacZ* reporter strains [26]. A 1 mL sample of WT or Δ*pvfA-D* culture supernatant was added to a 3 mL LSLB culture containing kanamycin and then inoculated with 10 μL of an overnight culture of the Δ*pvfC*::attTn7-P*mnl*-*lacZ* reporter strain. Two biological replicates were prepared from each supernatant sample. A 1 mL sample of LSLB was added as a control to the reporter strains, Δ*pvfC*::attTn7-P*mnl*-*lacZ* and WT::attTn7-P*mnl*-*lacZ*, instead of supernatant. Assay cultures were grown for 24 hr. At 24 hr, each β-gal assay culture was diluted 1:10 in 1 mL of fresh Z-buffer (60 mM Na_2_HPO_4_, 40 mM NaH_2_PO_4_, 10 mM KCl, 1 mM MgSO_4_, 50 mM 2-mercaptoethanol, pH 7.0) and the OD_600_ recorded. Samples were then further diluted in 1 mL of Z buffer as follows (two technical replicates were prepared from each sample)– 1:20 for the wildtype reporter strain control, 1:4 for the Δ*pvfC* reporter strain control, 1:20 for the Δ*pvfC* reporter strain complemented with wildtype supernatant, and 1:5 for the Δ*pvfC* reporter strain complemented with Δ*pvfA-D* supernatant.

The β-gal assay samples were then lysed with the addition of 100 μL chloroform and 50 μL 0.1% (w/v) SDS. Samples were briefly vortexed, then incubated for 5 min at room temperature. The reaction was started with the addition of 200 μL of fresh *ortho*-nitrophenyl-β-galactoside (ONPG) substrate (4 mg mL^-1^ in Z-buffer), then incubated at 28°C with shaking at 600 rpm. As soon as the reaction developed a yellow color, the reaction was moved to the benchtop and 500 μL of stop solution (1 M Na_2_CO_3_) immediately added. Reaction start and stop time was recorded. Absorbance at 420 nm and 550 nm was recorded using a BMG Labtech CLARIOstar *Plus* microplate reader. Promoter activity in Miller units (MU) was calculated as follows:

$$Millerunits=\frac{1000*(A420-1.75*A550)}{t*v*A600}$$

A420 = absorbance at 420 nm (yellow)

A550 = absorbance at 550 nm (background scattering)

A600 = absorbance at 600 nm (cell density)

t = time of reaction (min)

v = volume of cells in reaction (mL)

## Supporting information

S1 Fig Composition and signaling activity of *Pseudomonas virulence factor* (*pvf*) in *Pseudomonas entomophila* L48. (**A**) The *pvf* cluster contains four genes: *pvfB* (purple) encodes a non-heme diiron *N*-oxygenase, *pvfC* (blue) encodes a nonribosomal peptide synthetase, and *pvfA* (red) and *pvfD* (green) encode uncharacterized proteins. (**B**) *pvf*-encoded enzymes produce quorum sensing molecules (PVF autoinducers) that regulate the expression of many genes. Deletion of *pvfA*-*D* alters the expression of these genes.
(TIF)

S2 Fig Cultures used for RNA extraction exhibit the expected signaling activity. Signaling activity was indicated by the β-galactosidase activity in Miller Units (MU) of the P*mnl*-*lacZ* reporter strains, WT::P*mnl*-*lacZ* (WTrep) and Δ*pvfC*::P*mnl*-*lacZ* (Δ*pvfC*rep). Signaling activity of WTrep culture, Δ*pvfC*rep culture, and Δ*pvfC*rep cultures that were supplemented with the supernatant of cultures used for RNA extraction (left to right, WT1a-c, KO1a-c, WT2a-c, or KO2a-c).
(TIF)

S3 Fig Principal component analysis of RNA sequencing results. (**A**) Principal component analysis (PCA) of rlog transformed gene count table for each RNA sample. Group one corresponds to the first batch of RNA samples listed in Table 1 and S1 Table and group two corresponds to the second batch. WT (group one green; group two purple) and Δ*pvfA-D* (KO; group one red; group two blue) datasets form separate clusters. (**B**) PCA results for group two samples only. WT samples (purple) and Δ*pvfA-D* (KO) samples (blue) cluster separately.
(TIF)

S4 Fig Sequence alignment of VgrG proteins in *P*. *entomophila* L48. Sequence alignment was performed with Clustal Omega [83]. (**A**) Matrix of pairwise amino acid sequence identity between all VgrG proteins, shown as a percentage. Pairwise sequence identity comparisons *within* VgrG groups are color-coded for group 1 (blue), group 2 (pink), and other VgrGs (gold). Pairwise sequence identity comparisons *between* VgrG groups are color-coded for group 1 to group 2 (purple), group 1 to the VgrG of the Pent-T6SS locus (PSEEN0540, grey), group 1 to other VgrGs (green), group 2 to PSEEN0540 (grey), and group 2 to other VgrGs (orange). (**B**) Average pairwise amino acid sequence identity within and between VgrG groups. Color-coding is the same as (**A**). Error bars indicate standard deviation.
(TIF)

S5 Fig MultiGeneBlast results for unknown cluster 4 (PSEEN0621−PSEEN0624). (TIF)

S6 Fig MultiGeneBlast results for unknown cluster 5 (PSEEN0961−PSEEN0972). (TIF)

S7 Fig MultiGeneBlast results for unknown cluster 6 (PSEEN0973−PSEEN0987). (TIF)

S8 Fig MultiGeneBlast results for unknown cluster 7 (PSEEN2014−PSEEN2021). (TIF)

S9 Fig MultiGeneBlast results for unknown cluster 8 (PSEEN2139−2144, 2146–2156). (TIF)

S10 Fig MultiGeneBlast results for unknown cluster 9 (PSEEN2466−PSEEN2482). (TIF)

S11 Fig MultiGeneBlast results for unknown cluster 10 (PSEEN3319−PSEEN3331). (TIF)

S1 Table Summary of RNA sequencing results for all samples. (PDF)

S1 Dataset RNAseq complete results. (**Tab 1**) RNA sequencing results for all annotated genes in the *P*. *entomophila* genome. (**Tab 2**) All differentially expressed genes (*p* < 0.05, FC (WT/KO) > 2). (**Tab 3**) Differentially expressed genes involved in branched chain amino acid biosynthesis. (**Tab 4**) Differentially expressed genes involved in the type VI secretion system. (**Tab 5**) Differentially expressed genes involved in siderophore transport. (**Tab 6**) Differentially expressed virulence factor genes. (**Tab 7**) Differentially expressed genes involved in small molecule biosynthesis. (**Tab 8**) Raw results from DeSeq2. (**Tab 9**) Raw count table from featureCounts.
(XLSX)
