# Supplementary figures and images for: Pseudomonas virulence factor controls expression of virulence genes in Pseudomonas entomophila
Source: PLoS One. 2023 May 18;18(5):e0284907. doi: 10.1371/journal.pone.0284907 (PMC10194917; doi:10.1371/journal.pone.0284907)

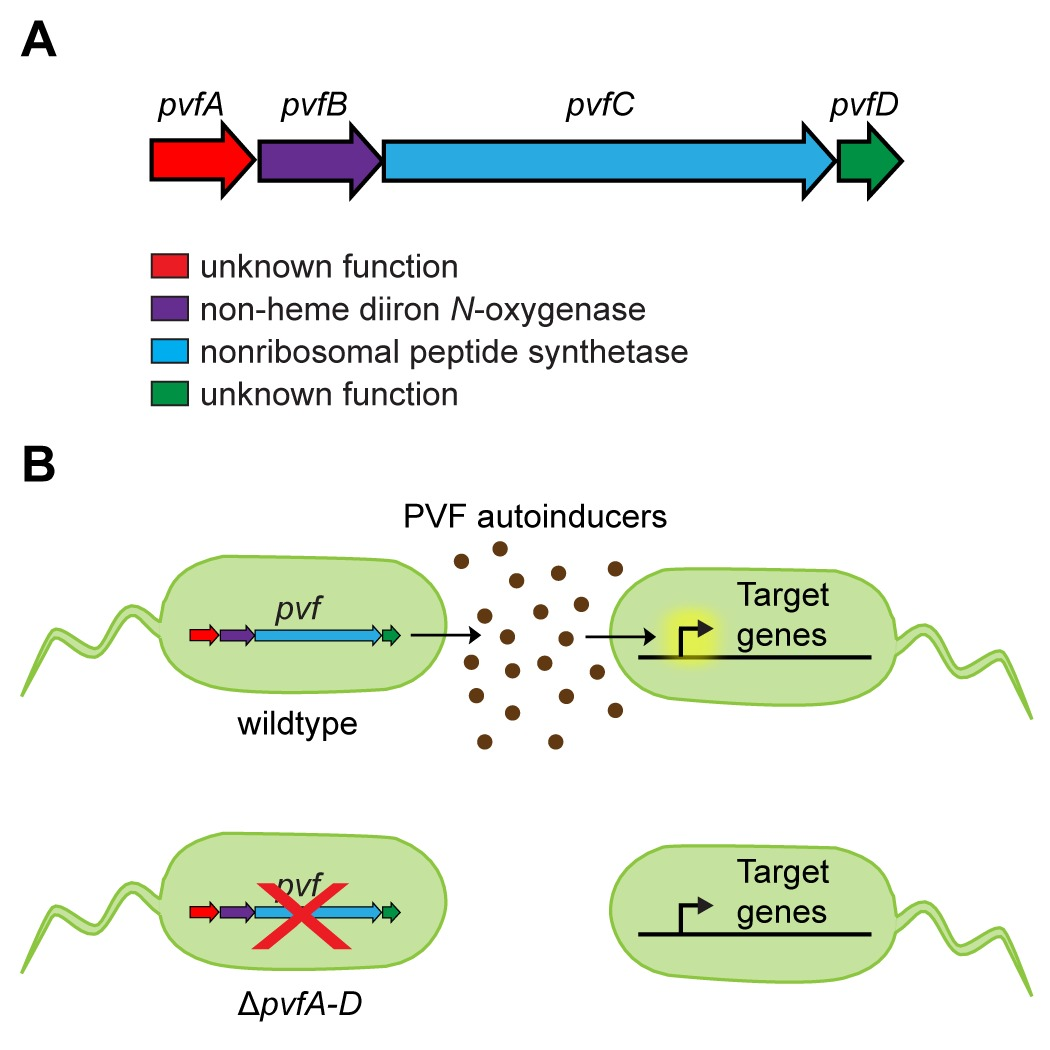

Supplement: S1 Fig — (A) The pvf cluster contains four genes: pvfB (purple) encodes a non-heme diiron N-oxygenase, pvfC (blue) encodes a nonribosomal peptide synthetase, and pvfA (red) and pvfD (green) encode uncharacterized proteins. (B) pvf-encoded enzymes produce quorum sensing molecules (PVF autoinducers) that regulate the expression of many genes. Deletion of pvfA-D alters the expression of these genes. (TIF) [file pone.0284907.s001.tif]

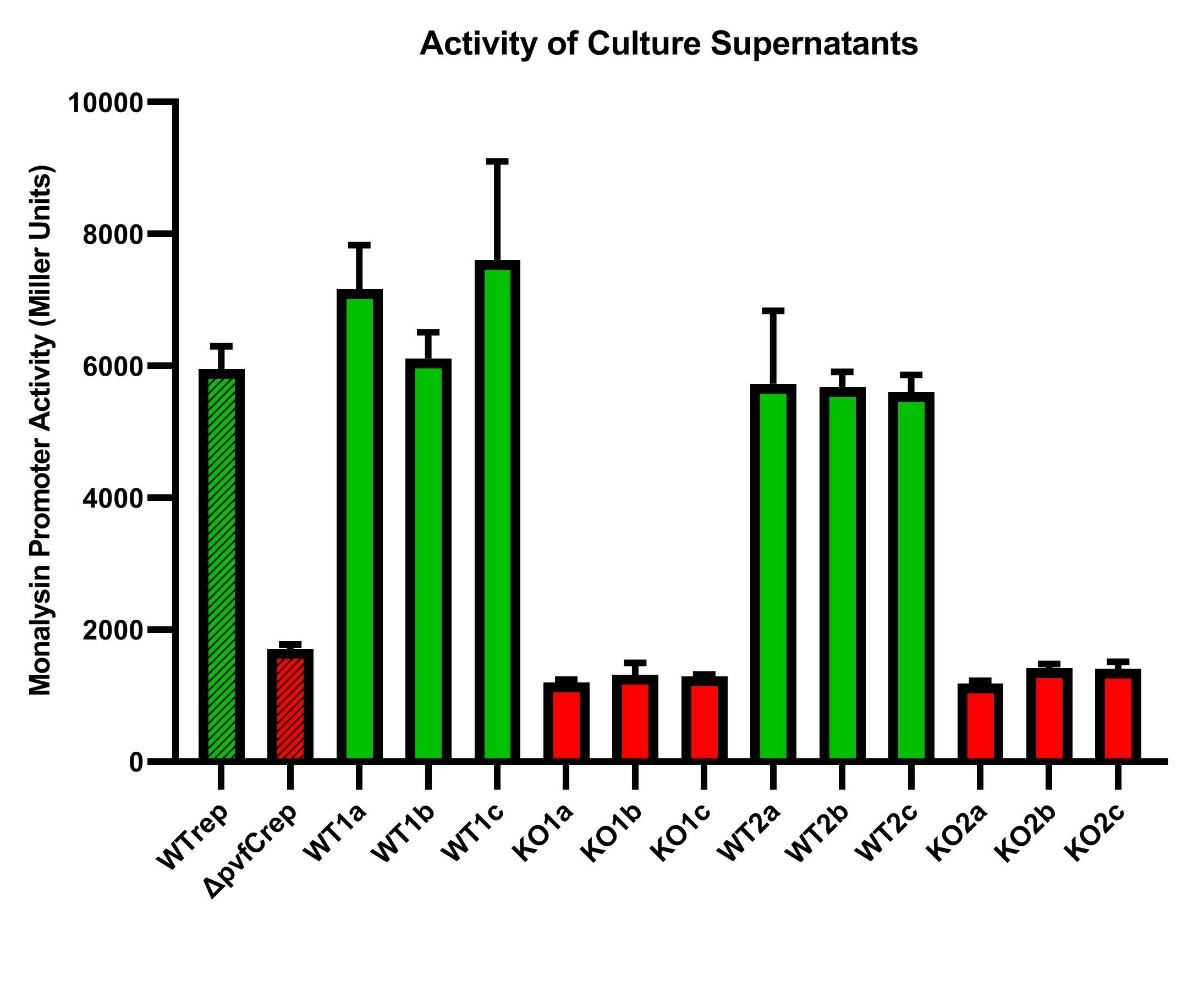

Supplement: S2 Fig — Signaling activity was indicated by the β-galactosidase activity in Miller Units (MU) of the Pmnl-lacZ reporter strains, WT::Pmnl-lacZ (WTrep) and ΔpvfC::Pmnl-lacZ (ΔpvfCrep). Signaling activity of WTrep culture, ΔpvfCrep culture, and ΔpvfCrep cultures that were supplemented with the supernatant of cultures used for RNA extraction (left to right, WT1a-c, KO1a-c, WT2a-c, or KO2a-c). (TIF) [file pone.0284907.s002.tif]

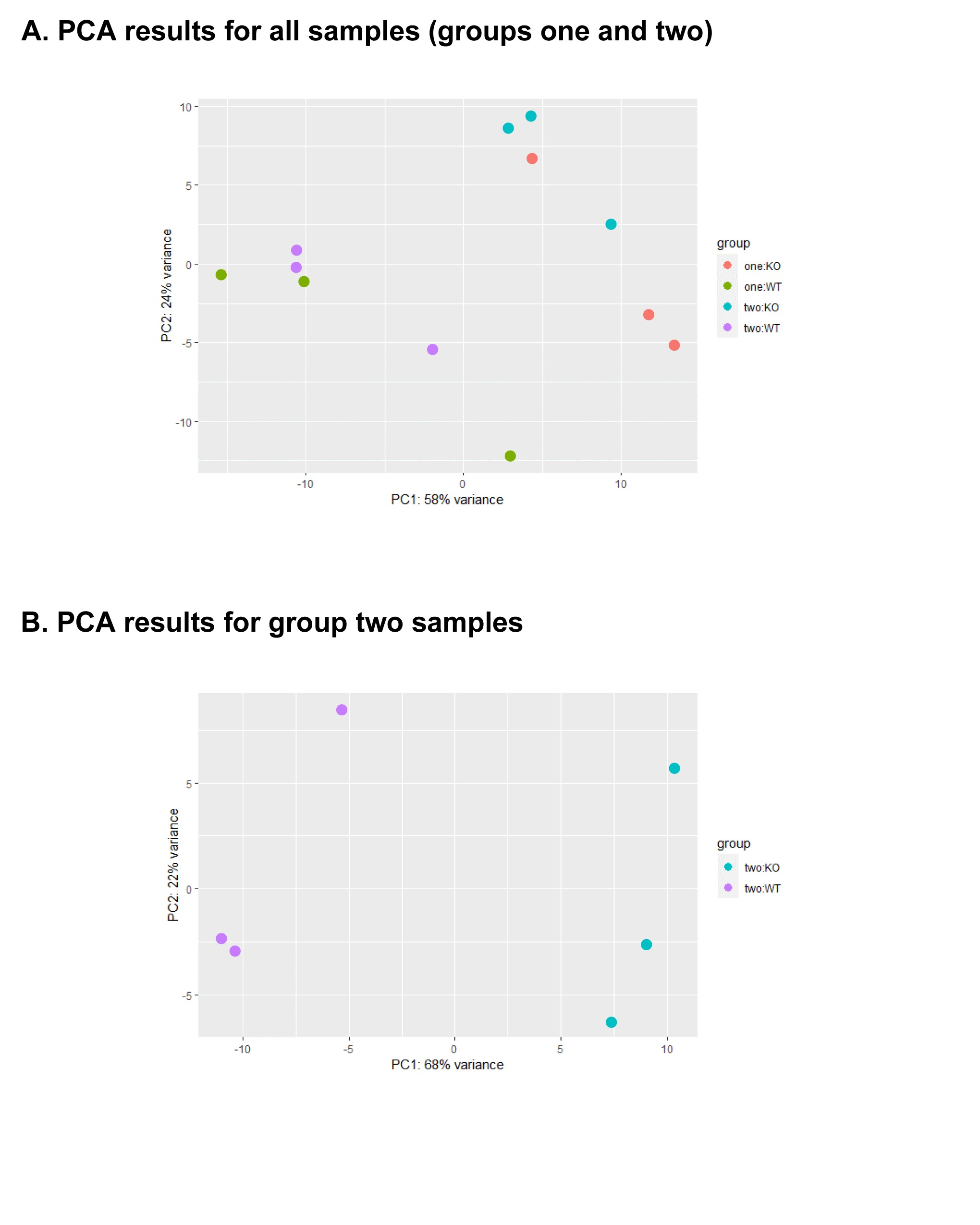

Supplement: S3 Fig — (A) Principal component analysis (PCA) of rlog transformed gene count table for each RNA sample. Group one corresponds to the first batch of RNA samples listed in Table 1 and S1 Table and group two corresponds to the second batch. WT (group one green; group two purple) and ΔpvfA-D (KO; group one red; group two blue) datasets form separate clusters. (B) PCA results for group two samples only. WT samples (purple) and ΔpvfA-D (KO) samples (blue) cluster separately. (TIF) [file pone.0284907.s003.tif]

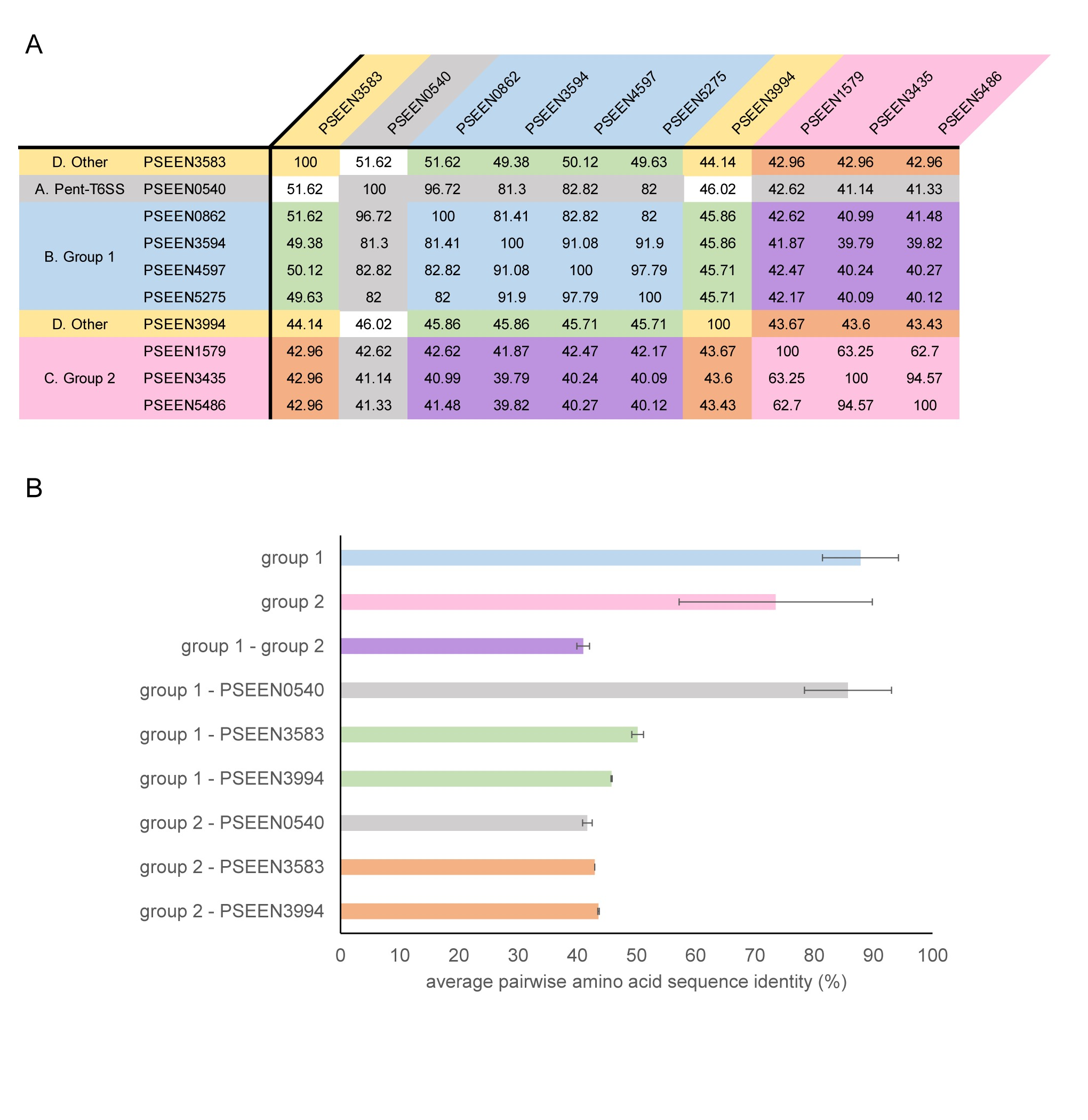

Supplement: S4 Fig — Sequence alignment was performed with Clustal Omega [83]. (A) Matrix of pairwise amino acid sequence identity between all VgrG proteins, shown as a percentage. Pairwise sequence identity comparisons within VgrG groups are color-coded for group 1 (blue), group 2 (pink), and other VgrGs (gold). Pairwise sequence identity comparisons between VgrG groups are color-coded for group 1 to group 2 (purple), group 1 to the VgrG of the Pent-T6SS locus (PSEEN0540, grey), group 1 to other VgrGs (green), group 2 to PSEEN0540 (grey), and group 2 to other VgrGs (orange). (B) Average pairwise amino acid sequence identity within and between VgrG groups. Color-coding is the same as (A). Error bars indicate standard deviation. (TIF) [file pone.0284907.s004.tif]

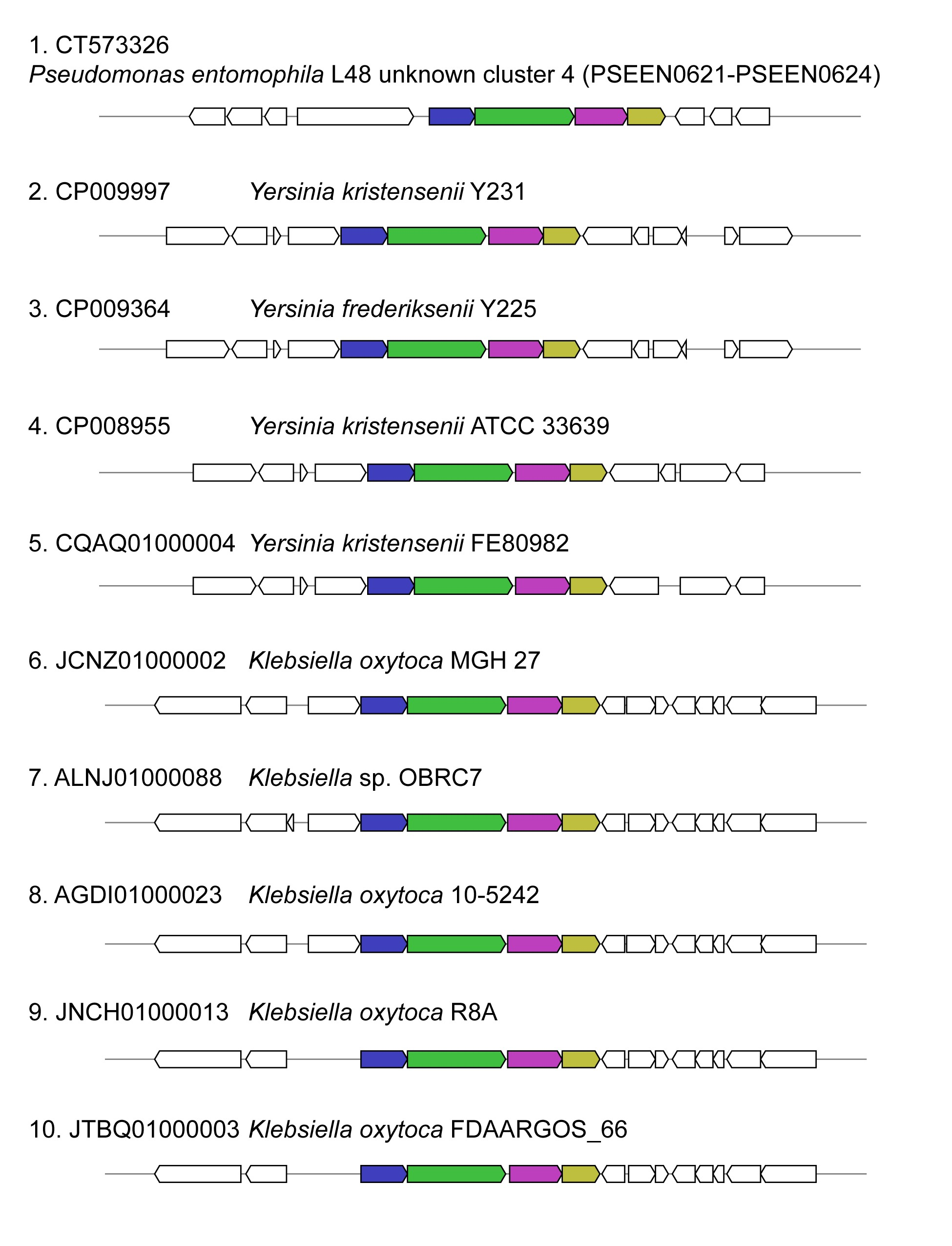

Supplement: S5 Fig — (TIF) [file pone.0284907.s005.tif]

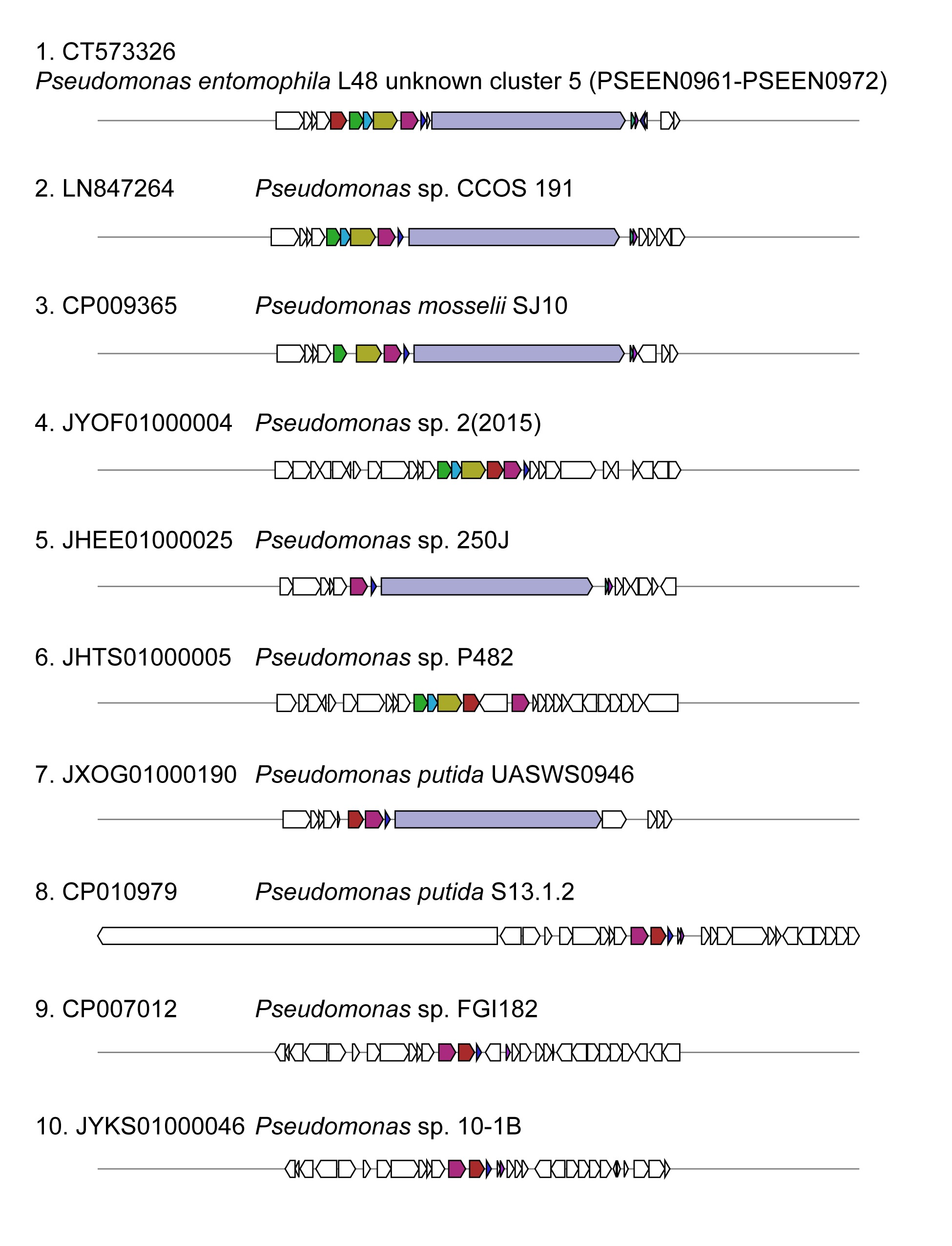

Supplement: S6 Fig — (TIF) [file pone.0284907.s006.tif]

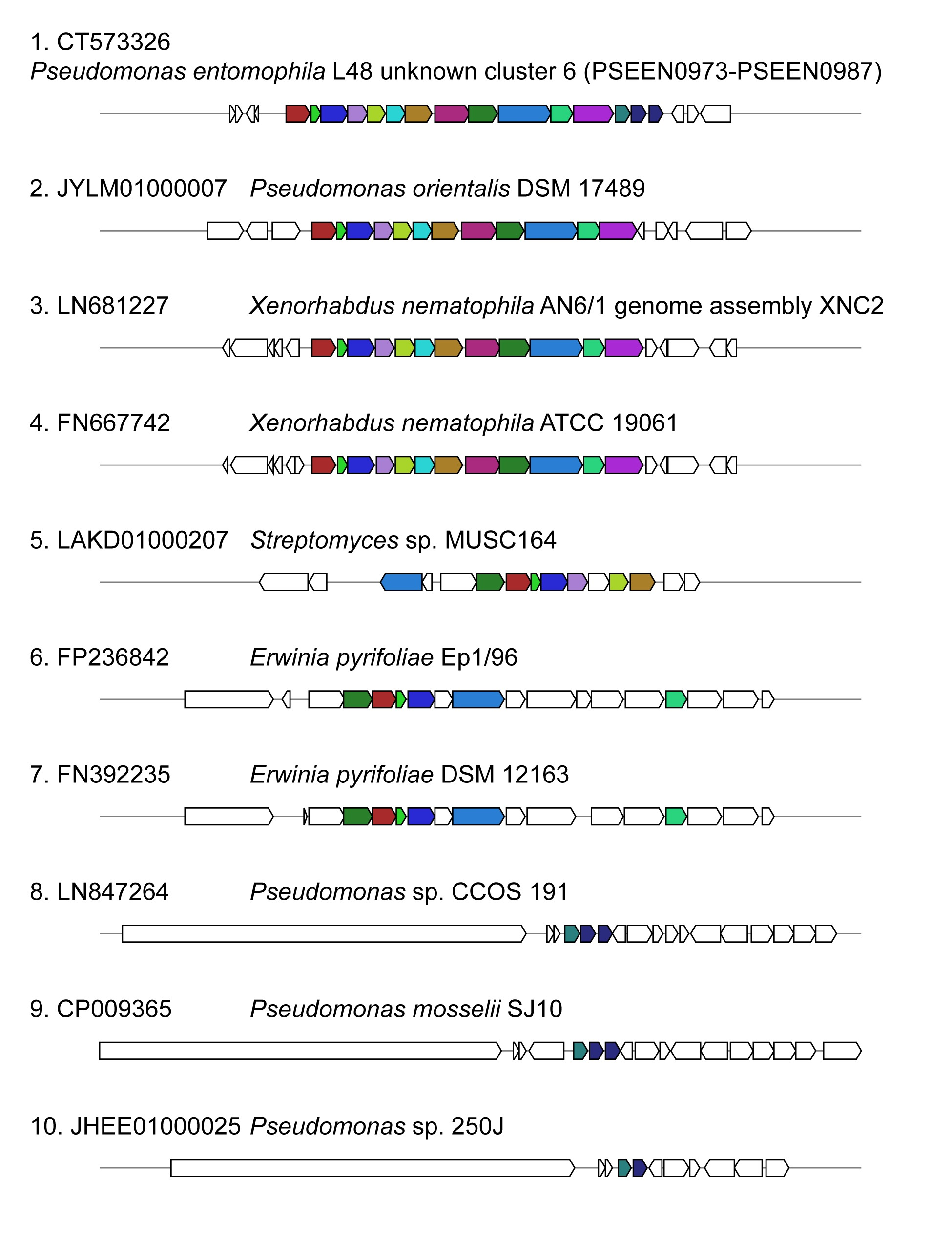

Supplement: S7 Fig — (TIF) [file pone.0284907.s007.tif]

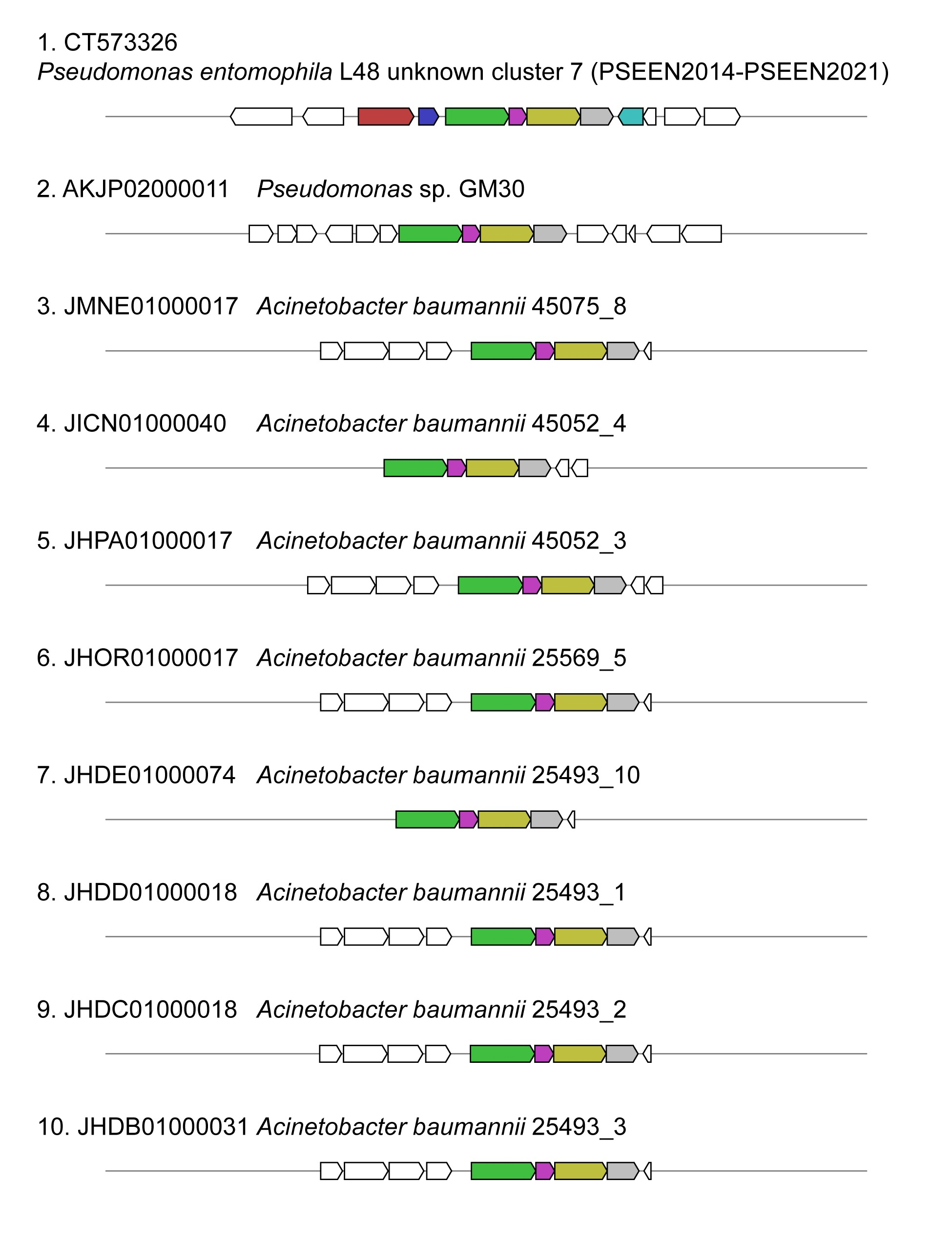

Supplement: S8 Fig — (TIF) [file pone.0284907.s008.tif]

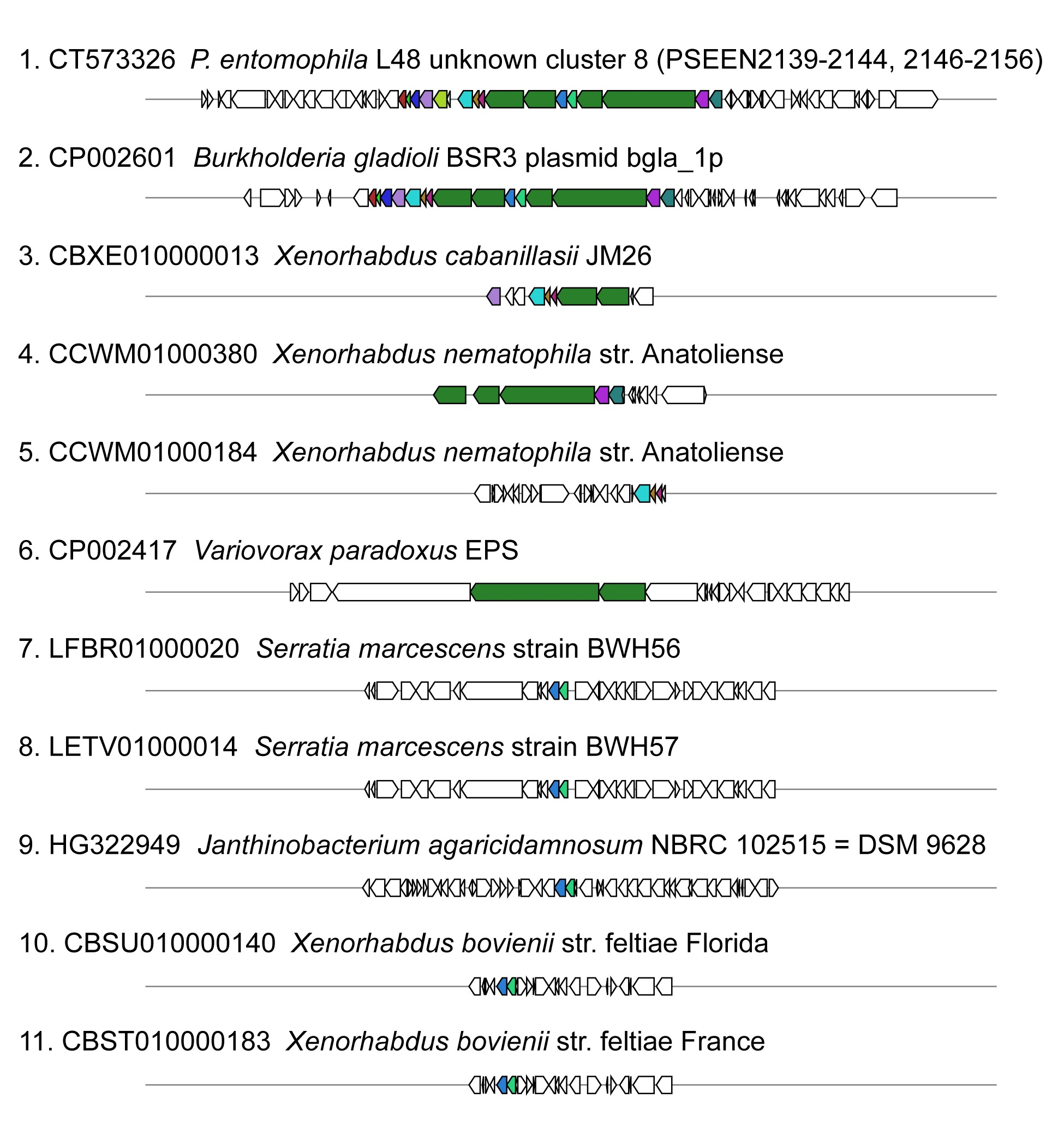

Supplement: S9 Fig — (TIF) [file pone.0284907.s009.tif]

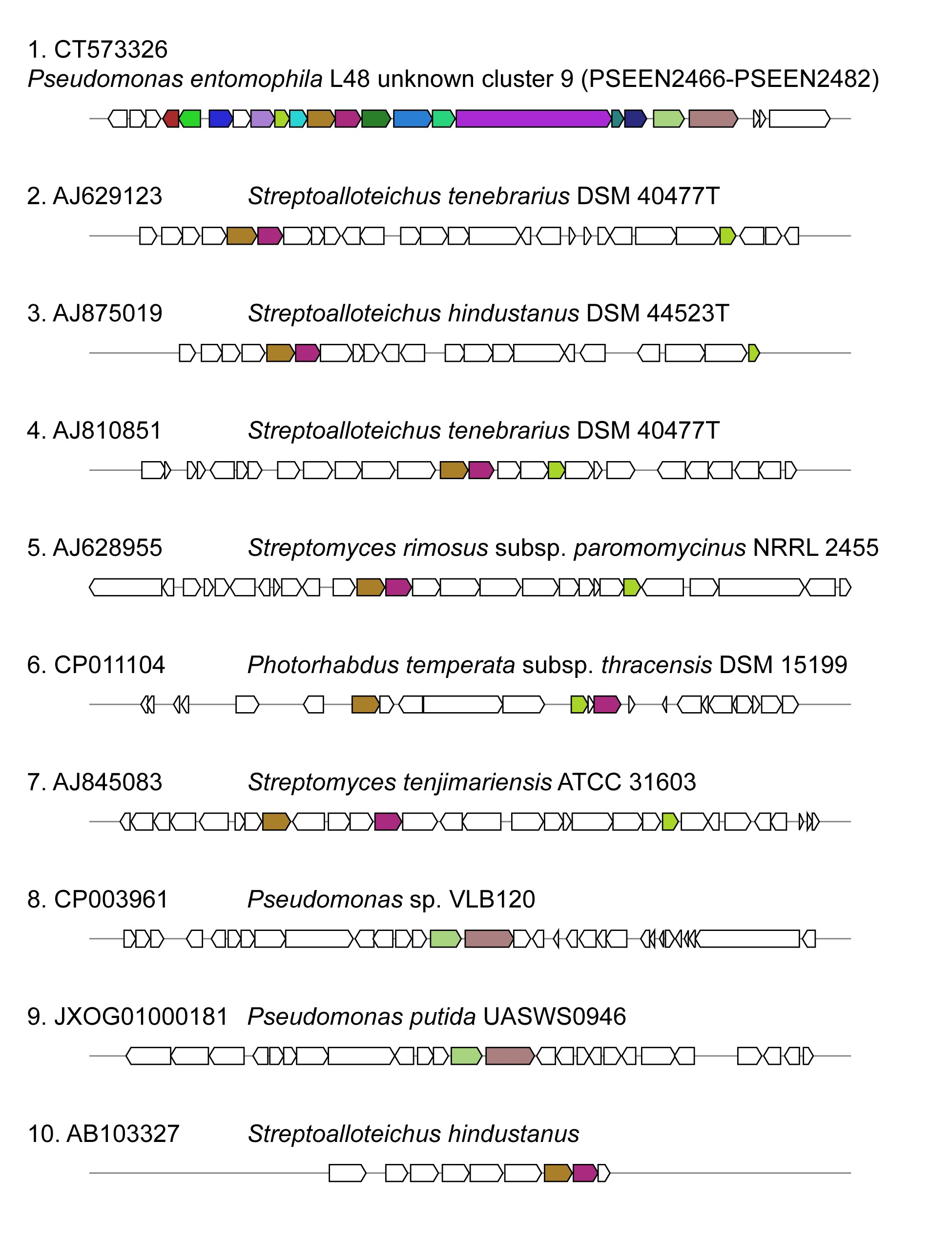

Supplement: S10 Fig — (TIF) [file pone.0284907.s010.tif]

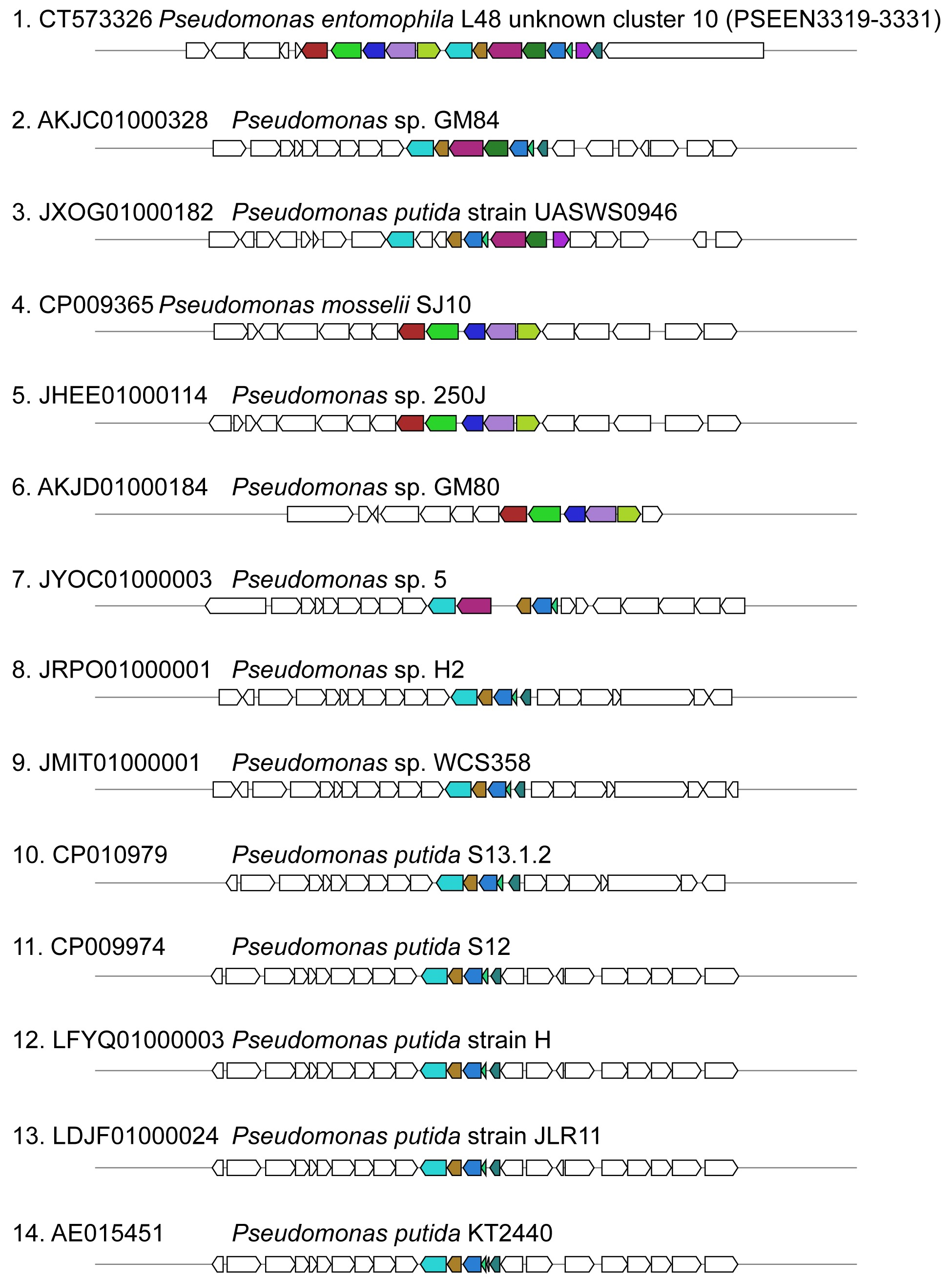

Supplement: S11 Fig — (TIF) [file pone.0284907.s011.tif]
