# Supplementary material for: Pseudomonas virulence factor controls expression of virulence genes in Pseudomonas entomophila
Source: PLoS One. 2023 May 18;18(5):e0284907. doi: 10.1371/journal.pone.0284907 (PMC10194917; doi:10.1371/journal.pone.0284907)
Supplement: S1 Table — (PDF) [file pone.0284907.s012.pdf]

S3 Table. Summary of RNA sequencing results for all samples

Sample number*	Q20 (%)	GC (%)	Total reads	Total mapped reads[a]	CDS mapped reads[b]	rRNA mapped reads[b]	tRNA & misc. RNA mapped reads[b]
1 – WT [1]	97.9	59.4	31,696,112	30,625,484 (96.6%)	16,893,160 (55.2%)	133,708 (0.4%)	13,598,616 (44.4%)
2 – WT [1]	97.7	58.9	30,817,242	29,824,354 (96.8%)	15,048,332 (50.5%)	132,590 (0.4%)	14,643,432 (49.1%)
3 – WT [1]	96.0	59.0	21,125,676	20,554,070 (97.3%)	9,674,456 (47.1%)	86,210 (0.4%)	10,793,404 (52.5%)
4 – KO [1]	97. 8	58.5	30,115,144	29,149,570 (96.8%)	13,554,234 (46.5%)	158,086 (0.5%)	15,437,250 (53.0%)
5 – KO [1]	97.8	59.1	24,199,096	23,401,150 (96.7%)	11,915,190 (50.9%)	155,544 (0.7%)	11,330,416 (48.4%)
6 – KO [1]	97.9	58.7	23,429,240	22,715,842 (97.0%)	10,956,838 (48.2%)	117,458 (0.5%)	11,641,546 (51.2%)

Q20, quality score. GC, total GC content of sample. CDS, DNA protein coding sequence.

* Numbers in brackets indicate batch number ([1] or [2]).

[a] Percentage shown per sample is total mapped reads over total reads.

[b] Percentage shown per sample is reads mapped to CDS, rRNA, or tRNA & miscellaneous RNA sequences over total mapped reads.

S3 Table. continued

Sample number*	Q20 (%)	GC (%)	Total reads	Total mapped reads[a]	CDS mapped reads[b]	rRNA mapped reads[b]	tRNA & misc. RNA mapped reads[b]
7 – WT [2]	97.6	59.0	28,490,164	27,489,494 (96.5%)	14,646,712 (53.3%)	115,864 (0.4%)	12,726,918 (46.3%)
8 – WT [2]	97.5	59.0	31,395,874	30,364,322 (96.7%)	15,685,992 (51.7%)	98,584 (0.3%)	14,579,746 (48.0%)
9 – WT [2]	97.8	58.8	30,696,600	29,676,324 (96.7%)	14,386,366 (48.5%)	82,002 (0.3%)	15,207,956 (51.2%)
10 – KO [2]	97.9	58. 8	30,466,038	29,174,436 (95.8%)	13,954,496 (47.8%)	105,352 (0.4%)	15,114,588 (51.8%)
11 – KO [2]	97.8	58. 7	30,684,052	29,666,404 (96.7%)	13,822,036 (46.6%)	128,546 (0.4%)	15,715,822 (53.0%)
12 – KO [2]	97. 8	58.7	30,044,730	29,094,042 (96.8%)	13,409,494 (46.1%)	135,672 (0.5%)	15,548,876 (53.4%)

Q20, quality score. GC, total GC content of sample. CDS, DNA protein coding sequence.

* Numbers in brackets indicate batch number ([1] or [2]).

[a] Percentage shown per sample is total mapped reads over total reads.

[b] Percentage shown per sample is reads mapped to CDS, rRNA, or tRNA & miscellaneous RNA sequences over total mapped reads.
